# Meiotic DNA breaks drive multifaceted mutagenesis in the human germline

**DOI:** 10.1126/science.adh2531

**Published:** 2023-12-01

**Authors:** Robert Hinch, Peter Donnelly, Anjali Gupta Hinch

**Affiliations:** 1 Big Data Institute, University of Oxford; Oxford, UK; 2 Wellcome Centre for Human Genetics, University of Oxford; Oxford, UK; 3 Genomics plc; Oxford, UK

## Abstract

Meiotic recombination commences with hundreds of programmed DNA breaks, however the degree to which they are accurately repaired remains poorly understood. We report that meiotic break repair is 8-fold more mutagenic for single-base substitutions than was previously understood, leading to de novo mutation in 1 in 4 sperm and 1 in 12 eggs. Its impact on indels and structural variants is even higher, with 100-1300-fold increases in rates per break. We uncover novel mutational signatures and footprints relative to break sites, which implicate unexpected biochemical processes and error-prone DNA repair mechanisms including translesion synthesis and end-joining in meiotic break repair. We provide evidence that these mechanisms drive mutagenesis in human germlines and lead to disruption of hundreds of genes genome-wide.

Meiotic recombination is essential for creation of gametes in most sexually reproducing species (1). It shuffles genetic material and, together with mutation, creates all genetic diversity. Recombination is initiated by induction of hundreds of programmed DNA double-strand breaks (DSBs) (2, 3). In many vertebrates, including humans, these breaks cluster into narrow regions of the genome, called “hotspots”, which are bound by the protein PRDM9 in a sequence-specific manner (4–6) (Fig. 1A). DNA is cut by SPO11, which is followed by resection, which generates 2-4 kb of single-stranded DNA (ssDNA) per break on average. In humans, a relatively small number of breaks (~45-60 in males and ~80-95 in females) are repaired with a crossover (7, 8). Most breaks are repaired without a crossover through a distinct homologous recombination (HR) pathway, also using the homologous chromosome as template (9). This leads to short segments of DNA being copied from the homologous chromosome, known as non-crossovers. Any remaining breaks are thought to be repaired using HR with the sister chromatid (1). Other repair pathways that are utilized in non-meiotic cells, such as end-joining, are thought to be suppressed in meiosis (10). Nevertheless, end-joining can occur during meiosis in mutant mice that lack a critical DNA repair protein (11) or when DSBs are induced by radiation (12).

Recombination and mutation are traditionally thought of as distinct mechanisms generating genetic variation. Nevertheless, work in yeast has shown that meiotic cells accumulate more mutations than mitotic cells (13, 14) in a SPO11-dependent manner (15) (reviewed in (16)). Studies in non-meiotic systems in bacteria and yeast have shown that repair of DNA breaks is mutagenic (17, 18) (reviewed in (19)). Although there are major differences between meiotic and non-meiotic break repair (1), flawed repair is a likely cause of mutagenesis in yeast meiosis (16).

In humans, indirect population-based approaches have shown an excess of rare C>G, A>G and C>T single nucleotide polymorphisms (SNPs) in recombination hotspots (20), with C>G elevation in specific scenarios (e.g. aging oocytes (21, 22), X chromosome in males (23)). Structural variants (SVs), defined as polymorphisms impacting 50+ bp of DNA are over-represented in hotspots (24). Minisatellite repeat instability (24–26) and genome rearrangements between regions of high sequence homology can occur from flawed recombination (27, 28). A recent paper leveraged the extensive pedigree information in Iceland to establish that de novo single-base substitutions are enriched near crossovers (7), consistent with findings from sperm-typing (29).

Despite these developments, the provenance of mutations associated with meiotic breaks remains poorly understood across all size scales, especially in higher eukaryotes. The burden of single-base substitutions due to the vast majority (~90%) of DSBs, i.e., those not repaired with a crossover, is unknown. Indels (defined as insertions or deletions less than 50bp long) in unique DNA remain unexplored. The nature, provenance, and impact of SVs due to this process also remain uncharacterised in general.

To address these questions, we have harnessed a range of population-scale resources to construct detailed base-pair resolution maps of mutation relative to human recombination hotspots. These data include de novo and extremely rare genetic variation, comprising 341 million SNPs, 64 million short indels, and 0.5 million SVs. These high-resolution maps enable us to characterize sequence properties of mutations and compare their footprints with the localization of distinct molecular processes taking place within hotspots (Fig. 1A). They reveal the scale of mutagenesis and link it with particular DNA repair processes, thereby providing novel insights on the nature, impacts, and mechanisms of these errors in the human germline.

## Burden of de novo single-base substitutions due to meiotic break repair is 8-fold higher than previously understood

Unlike crossovers, other repair outcomes such as non-crossovers are either difficult or impossible to detect in gametes or pedigrees (9, 30, 31). As a result, their mutational impact is unknown despite comprising ~90% of DSB outcomes (8, 9, 31–35). To solve this problem, we took an indirect approach: we measured the number of de novo mutations (DNMs) in recombination hotspots whilst controlling rigorously for local mutation rates. Recombination hotspots vary according to the DNA binding specificities of their PRDM9 alleles, with the so-called “A-like” alleles being the most common (36, 37). We identified the locations of 28,286 human recombination hotspots at base-pair resolution, by applying our published methodology (38) to published chromatin immunoprecipitation followed by ssDNA sequencing data measuring the occupancy of a key meiotic DSB repair protein (DMC1) in testes of an individual homozygous for the A-allele of PRDM9 (20, 39). DMC1 hotspots have similar localisation in males and females (40) and our approach is robust to sex differences in hotspot intensities (41). These hotspots comprise ~2% of the genome.

**Figure 1:**
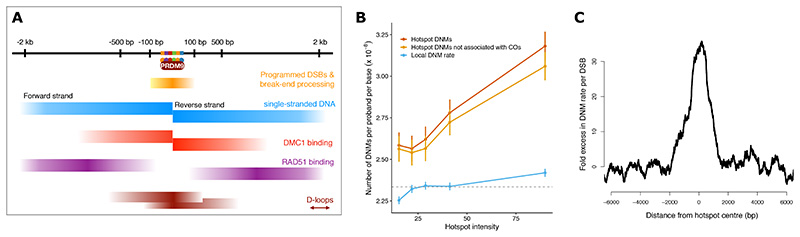
De novo mutations in human recombination hotspots. **(A)** Hotspots have a precise anatomy with zones in which distinct biochemical processes take place (not drawn to scale). PRDM9 binding is followed by induction of programmed DNA double-strand breaks (DSBs) by SPO11. SPO11 is released from break-ends and resection degrades one strand of DNA in the 5’ to 3’ direction generating 1-2 kb of single-stranded DNA on each side of break, which is to the left of the DSB on the forward strand and to its right on the reverse strand. These are bound by the key repair proteins DMC1 and RAD51, with DMC1 binding close to the DSB site and RAD51 away from it. DNA repair with the homologous chromosome is mediated through transient DNA structures called D-loops, which have the capacity to migrate. **(B)** Autosomal hotspots (n=25,440) were sorted by their DMC1 intensity and divided into 5 equal bins. The average number of paternal and maternal DNMs in hotspots per proband per base in each bin is shown: all DNMs within 1.5 kb of hotspot centres (red), DNMs within 1.5 kb of hotspot centres but not associated with a crossover (orange) and DNMs between 5 kb to 20 kb from hotspot centres (blue; rescaled for a 3 kb window to facilitate comparison). **(C)** Only a small proportion of hotspots (fewer than 1%) experience a break in any given meiosis. Here we show the DNM rate per break inferred from the enrichment of DNMs in hotspots (3 kb moving window). The centre of a hotspot is defined as the midpoint of its PRDM9 binding motif.

For each hotspot, we counted de novo single-base substitutions identified in 2,976 Icelandic trios (7) that were within 1.5kb of the hotspot center, as defined by the midpoint of its PRDM9 binding site. The number of DNMs was correlated with a measure of hotspot intensity (p=10^-13^, Table S1, (41)). However, the vast majority of DNMs were not associated with a crossover (Fig. 1B). Note that not all mutations in hotspots will be due to recombination (Fig. 1B) and will be impacted by local factors (e.g., GC-content (42)). To calculate the number of DNMs that are due specifically to the recombination machinery in each parent, we therefore accounted for local mutation rates and other factors (Fig. S1A-B, (41)). When restricted only to positions at which crossovers occurred in particular meioses, this approach gave similar mutation rate estimates to previously reported direct measurements (7), which provides validation of our indirect method.

The sex-specific DNM rates due to the recombination machinery (including all repair outcomes) we inferred are 0.234 (95% CI=[0.180,0.288]) DNMs per paternal and 0.080 (95% CI=[0.048,0.114]) DNMs per maternal meiosis. These are 8-fold higher than the mutation burden per meiosis due to crossovers alone in both males and females (the contribution of crossovers is 0.028 (95% CI=[0.022, 0.034]) for males and 0.011 (95% CI=[0.005,0.013]) for females in these hotspots). They comprise ~0.5% of the total burden of DNMs genome-wide and imply that, on average, ~1 in 4 sperm and ~1 in 12 eggs has a DNM specifically due to the meiotic recombination machinery.

The proportion of DNMs due to crossover (~12% for males and ~13% for females) are comparable to the proportion of DSBs that are resolved as crossover. These data support the parsimonious view that single-base substitutions in hotspots are driven by DSBs, and are not strongly influenced by the particular repair outcome. Since the number of crossovers per meiosis is well understood (7), we can estimate the average number of DSBs per meiosis under this model (41). These are 441 (95% CI=[327,590]) for males and 620 (95% CI=[349,1045]) for females. The inferred sex-averaged DNM rate per break is shown in Fig. 1C.

We estimated that the single-base substitution rate per programmed DSB is 6.6x10^-4^ in males and 2x10^-4^ in females on average (41). This implies that break repair is more error-prone in males, with mutation rate per break in male meiosis being ~3-fold higher than that in female meiosis. It is noteworthy that DNMs in the rest of the genome also occur ~3-4 times as often on the paternal relative to the maternal genome (7, 43).

## Footprints and mechanisms of single-base substitutions in hotspots

DNA breaks and a succession of repair processes occur within distinct segments inside recombination hotspots (Fig. 1A). PRDM9 binds a sequence motif at the center of hotspots and SPO11 induces breaks within ~100 bp from it. DNA on either side of the break undergoes 1-2 kb of resection to produce single-stranded DNA, which is bound by repair proteins DMC1 and RAD51 (Fig. 1A). Re-synthesis of DNA closest to the break site occurs within the context of a critical recombination intermediate known as the D-loop (Fig. 1A). It is not known how the remaining DNA, which comprises most of the resected segment, is repaired for the vast majority of breaks.

A natural question therefore is whether mutation rates and outcomes are different in these distinct segments of hotspots, the answer to which may further our understanding of the underlying mechanisms and repair processes. However, the resolution even with thousands of trios is insufficient to distinguish mutations in these segments (Fig. 1C). To overcome this problem, we leveraged extremely rare polymorphisms as a proxy for DNMs. This approach effectively includes DNMs that have arisen relatively recently in humans and the impact of selection and drive on them is expected to be minimal (23, 44). We used whole-genome polymorphism data provided by gnomAD, comprising over 70,000 genomes from several global populations (45). We filtered for high quality variants with allele frequency <10^-3^, which leaves 341 million single-base substitutions. This represents ~1800-fold increase in number of mutations relative to the trios above.

**Figure 2:**
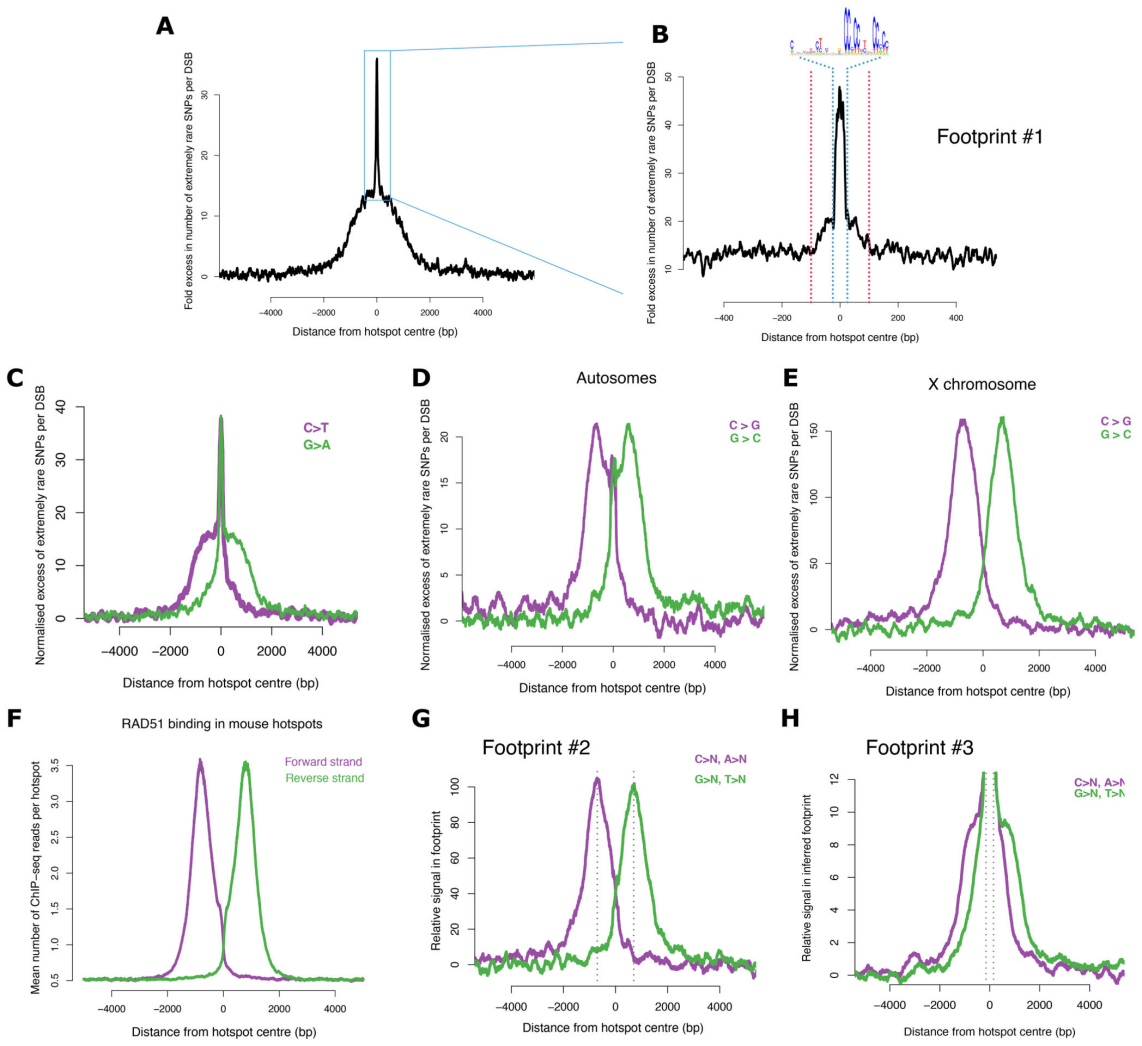
Footprints of extremely rare single-base substitutions relative to human hotspots. **(A)** Fold excess in the number of single-base substitutions (allele frequency < 10^-3^; henceforth, extremely rare) per DSB in and around autosomal hotspots (n=25,440). Each hotspot is centered at its inferred PRDM9 binding site (50 bp moving window). **(B)** Zoomed in view of (A) with 10 bp moving window. The PRDM9 binding site is highlighted (blue dotted lines). The motif may be present in the orientation shown or its reverse complement. Red dotted lines show ±100 bp from the motif centre. **(C)** As (A), but showing C>T and G>A mutations per ‘C’ and ‘G’ base, respectively (100 bp moving window). The figure corrects for differences in sequence composition in and around hotspots. **(D)** As (C), but showing C>G and G>C mutations per ‘C’ and G base, respectively (200 bp moving window) **(E)** As (D) but for the X chromosome **(F)** DNA binding footprint of RAD51 in mouse hotspots measured via ChIP-seq for RAD51 in mouse testes (47). **(G)** As (E) but combining single-base substitutions that exhibit ‘off-centre’ peaks of mutations, namely C>G, A>G, C>A, CpG>TpG, and their reverse complements on the X chromosome. **(H)** As (C) but for C>T (excluding CpG>TpG), A>T, A>C, and their reverse complements on the autosomes.

Mapping these mutations to hotspots reveals unexpected structure in the mutagenesis profile: a wide base of mutations ±2 kb from the hotspot centre (Fig. 2A), a peak of mutagenesis within 100 bp of the hotspot centre (Fig. 2A-B), and an additional intense peak within the PRDM9 binding site itself (Fig. 2B). Directly measured DSBs in mouse are also concentrated within ~100 bp from hotspots centres and have a further peak within the PRDM9 binding site (46).

Analysis of the mutation spectrum shows that all six single-base substitutions (C>T, C>G, C>A, A>G, A>C, A>T) and their reverse complements are enriched with distinctive footprints (Fig. 2C-E, S2A-E). Whereas DSBs impact both DNA strands, several downstream repair processes are strand-specific (Fig. 1A). As a result, mutations that arise due to strand-specific processes may exhibit strand “asymmetry”. For example, a mutation type may be enriched on one side of a break and its reverse complement on the other side. The presence or absence of strand asymmetry in mutations is thus informative about the nature of processes giving rising to them. We observe that mutations in the central ±100 bp do not exhibit strand asymmetry, which is consistent with them arising as a result of flawed processing of DNA break-ends (Fig. 1A). In contrast, mutations in the flanking 2kb exhibit strong strand asymmetry and are composed of C>N and A>N mutations on the forward strand and G>N and T>N mutations on the reverse strand (Fig. 2C-E, S2A-E).

Further analysis reveals distinct footprints within these flanks. Several mutation types (C>G, A>G, C>A) have “off-centre” peaks 700-800 bp from the centre (Fig. 2D-E, S2A,B,E). For C>G mutations on the X chromosome, for example, the off-centre peaks are particularly intense, subsuming any central signal (Fig. 2E). Strikingly, these peaks match the binding profile of the RAD51 recombinase in hotspots (Fig. 2F). Recent work in mouse (47) has shown that this is also the stretch of ssDNA that lies outside the “D-loop” (Fig. 1A), suggesting mechanism(s) involving DNA re-synthesis distal to the break-site. The footprint of the remaining mutations resembles localisation of ssDNA in hotspots, including ssDNA in the D-loop (Fig. 1A, 2H) and is consistent with hypermutation within it (48, 49).

In summary, our analyses have revealed unexpected complexity in mutagenesis, implicating three distinct factors, namely SPO11-mediated break machinery, ssDNA hypermutation, and DNA re-synthesis outside the D-loop, underlying the overall increase in single-base substitutions in hotspots. We investigate the mechanisms underlying these factors below.

## Hotspots are a significant source of indels and structural variants

To understand the role of meiotic breaks in generating length polymorphisms, we extended the approach above to indels and SVs. For indels, we used whole-genome data from gnomAD, comprising 64 million indels after filtering for variant quality. For SVs, we included the following datasets: (i) gnomAD-SV (387,780 SVs from 10,847 individuals from four global populations based on short-read data)(50) (ii) deCODE (133,886 SVs from 3,622 Icelanders based on long-read data)(24). The two SV datasets have contrasting strengths (size vs read length) and we use them as replication datasets.

**Figure 3:**
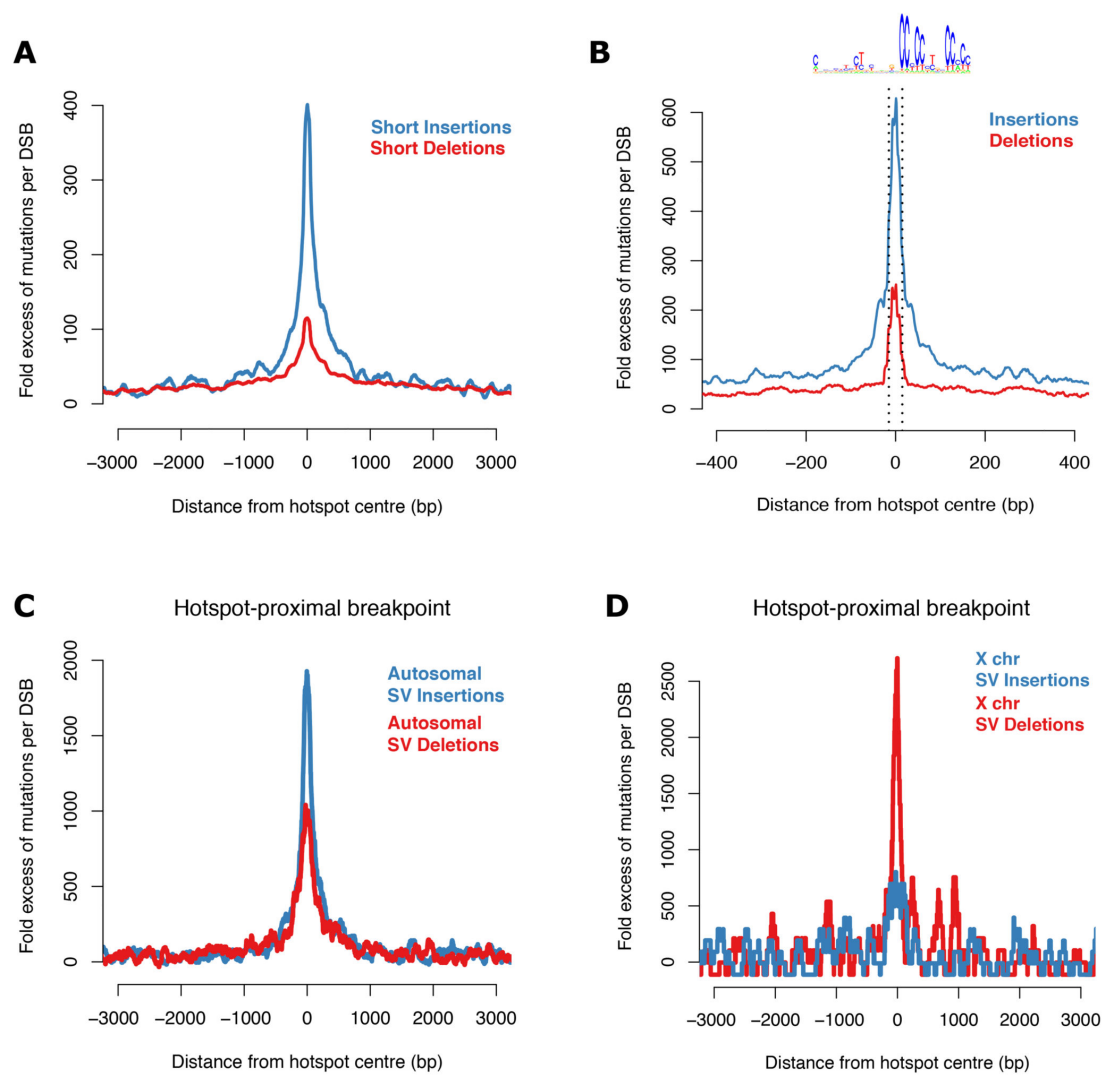
Footprints of extremely rare indel and SV breakpoints relative to human hotspots. **(A)** Fold excess in the number of indel breakpoints per DSB (allele frequency < 10^-3^) in and around autosomal hotspots (100 bp moving window). Indels overlapping Alu elements are not included and are shown separately in Fig. S6. Both breakpoints are included. **(B)** As (A) but a zoomed in view of 1 bp insertions and deletions (20 bp moving window). The PRDM9 binding site is highlighted. The motif may be present in the orientation shown or its reverse complement. **(C)** Fold excess in the number of SV breakpoints (allele frequency < 10^-2^) per DSB detected via long-read sequencing in an Icelandic population (deCODE-SV) relative to autosomal hotspots (100 bp moving window). The hotspot proximal breakpoint is shown. See Fig. S3 for the hotspot-distal breakpoint and data from multiple populations (gnomAD-SV). **(D)** As (C) but for the X chromosome (100 bp moving window).

First, we consider indels, which are insertions and deletions less than 50 bp long. Insertions are ~399-fold (95% CI=[395,403]) and deletions are 115-fold (95% CI=[113,117]) higher per break than would be expected in these regions in the absence of the break, i.e., from genomic insults collectively in the germline and the zygote (Fig. 3A). The footprint of 1 bp indels (Fig. 3B) shows strong concentration in the PRDM9 binding motif, similar to the footprint of single-base substitutions (Fig. 2B).

SVs are variants impacting 50+ bp of DNA and comprise mainly deletions and insertions (50)., Hotspots harbour one or both breakpoints of 7% of autosomal SVs. SV insertion breakpoints are 930-fold (95% CI=[848,1008]) and SV deletion breakpoints are 431-fold (95% CI=[376,488]) elevated per break, respectively (Fig. 3C, S3A-B). As is the case with (short) indels, SVs in autosomal hotspots are biased towards insertions.

Strikingly, the bias towards insertions is reversed on the X chromosome (Fig. 3D, Fig. S3C). Whilst there is an increase in SV insertions per break (243-fold, 95% CI=[43,456], hotspots on the X chromosome are a particularly significant source of SV deletions. They exhibit 1343-fold (95% CI=[885,1835]) increase in frequency per DSB (Fig. 3D) and harbour breakpoints of 10% of SV deletions on the X chromosome.

We characterise indel polymorphisms further below, stratifying them by the DNA context of their breakpoints (unique DNA or any of the known repetitive DNA families) to account for underlying differences in mutation propensity.

## Indels in hotspots are larger and are biased towards insertions in the autosomes

**Figure 4:**
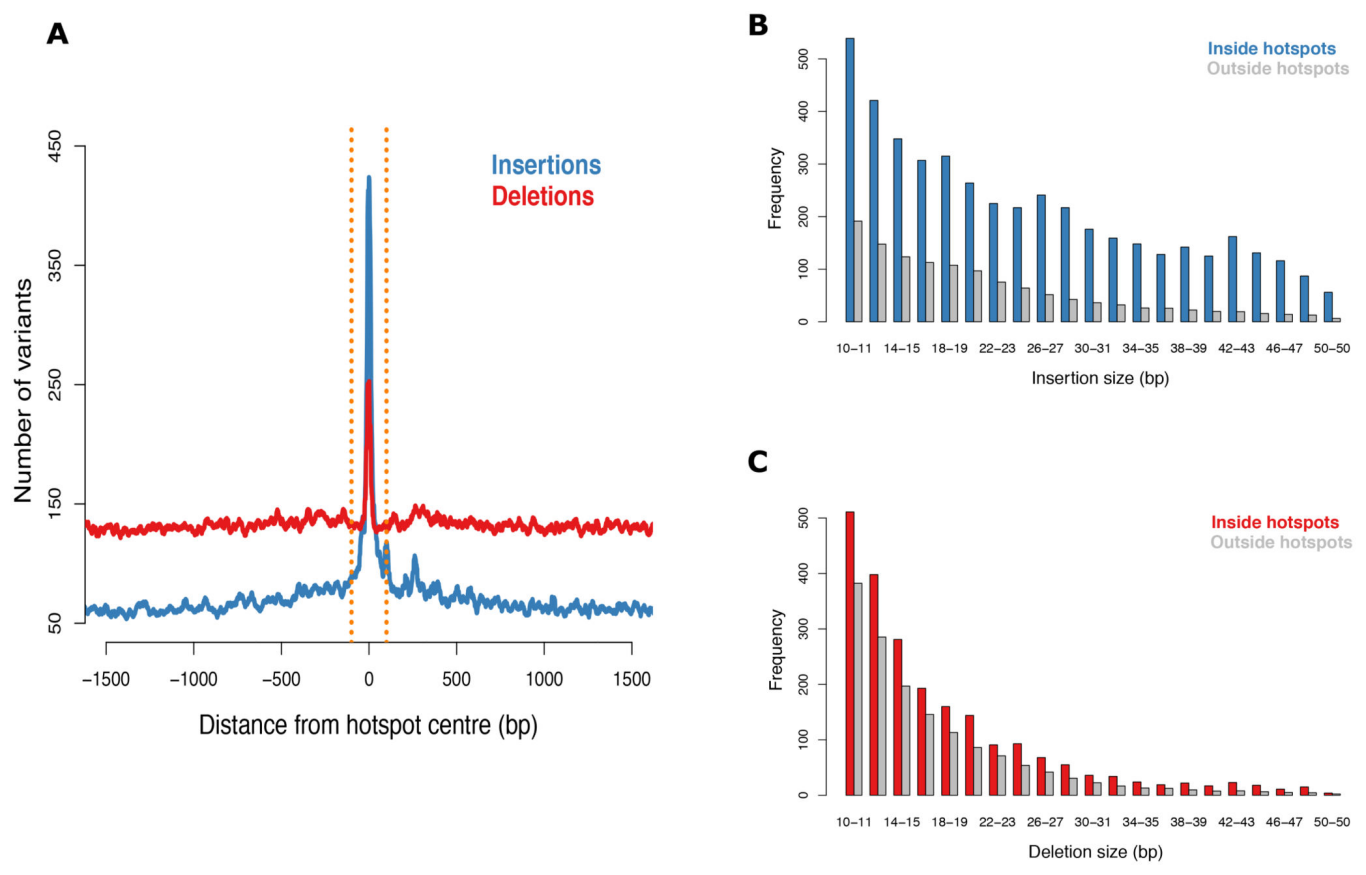
Properties of indels inside hotspots in unique DNA. **(A)** Number of insertions (blue) and deletions (red) in unique DNA in and around hotspots (20 bp moving window). Hotspot-proximal breakpoint is shown. **(B)** Histogram of number of insertions that have a breakpoint within 100 bp of hotspot centres (blue) or 8-10 kb from it (grey, rescaled to the average number per 200 bp to facilitate comparison). See Fig. S4A for the full size-scale. **(C)** As (B) but for deletions. See Fig. S4C for the full size-scale.

First we consider indels in unique DNA. Here, deletions outnumber insertions 2:1 outside autosomal hotspots (Fig. 4A). We observed the opposite inside hotspots, with a strong excess of insertions (Fig. 4A). Indels in hotspots are larger and more numerous than those outside (Fig. 4B-C, S4A-D). The number of insertions and deletions is correlated with hotspot intensity (p=2x10^-20^, p=9x10^-41^, for insertions and deletions, respectively, Fig. S4E-F). They are particularly elevated in hotspots close to telomeres (p=7x10^-8^, p=2x10^-11^, respectively) over and above the expectation from local mutation rates (as are single-base substitutions, Table S1). It is known that meiotic breaks lead to instability of minisatellites near telomeres (51). However, to the best of our knowledge, a higher mutation rate in repair of telomere-proximal breaks in general, including those in unique DNA, has not previously been reported.

Next we assess indels in Tandem Repeats (TRs). These have a different composition relative to those in unique DNA, both inside and outside hotspots. Insertion and deletion rates are similar outside hotspots, consistent with evolutionary stability of TR sequences on average (Fig. S5A). However, inside hotspots, there is a ~2-fold bias towards insertions relative to deletions (Fig. S5A) (25). Indels in hotspots are larger than those outside (Fig. S5B-E) and are usually multiples of the TR repeat-unit, particularly for insertions (Fig. S5F-G). Their rates are correlated with hotspot intensities and modulated by context (Table S1), including proximity to telomeres (Table S1) (51). Analysis of mutations in transposable elements provides further confirmation that elevated mutagenesis is due to the recombination machinery per se (as opposed to, say, sequence composition) (Fig. S6) (41).

We conclude that bias towards insertions and higher mutation rates near telomeres are a consistent feature of mutations arising from meiotic breaks in the autosomes.

## Disease impacts of mutations resulting from repair of meiotic breaks

**Figure 5:**
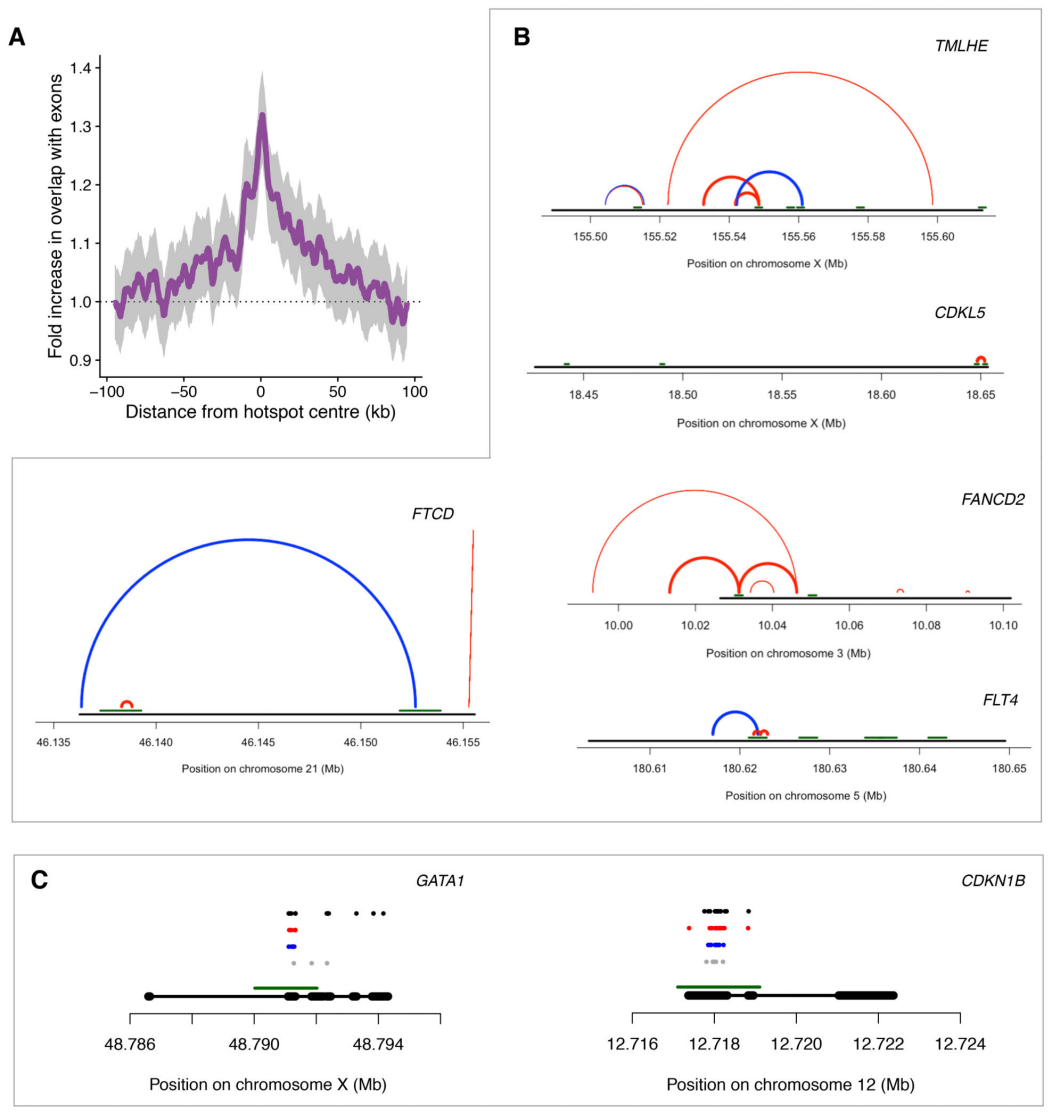
Disease impacts of mutations resulting from meiotic breaks. **(A)** Enrichment of exons near hotspots. For each hotspot, exons within 100 kb were included. Each base pair in an exon counts towards the total at the corresponding distance from each hotspot centre (3 kb moving window). 95% confidence intervals are shown in grey. **(B)** Examples of predicted loss-of-function SVs impacting genes associated with disease. Gene bodies (black lines), hotspots (horizontal green lines), insertions (blue arcs joining start and end points) and deletions (red arcs joining start and end points). SVs with breakpoints in hotspots shown with thicker arcs. Genes (and associated diseases) shown are: *TMLHE* (Autism X-linked) and *CDKL5* (Developmental and Epileptic Encephalopathy, Atypical Rett syndrome), *FTCD* (Glutamate Formiminotransferase Deficiency), *FANCD2* (Fanconi Anaemia), and *FLT4* (Lymphatic Malformation, Congenital Heart Defects) **(C)** As (B) but for ClinVar data. Exons (thick black lines), hotspots (horizontal green lines) and reported pathogenic mutations are shown, which are single-base substitutions (black), deletions (red), insertions (blue), other or unspecified (grey). Genes shown are *GATA1* (Thrombocytopenia, Thalassemia) and *CDKN1B* (Neoplasia).

Exons are over-represented near hotspots for the PRDM9 alleles mapped in humans (Fig. 5A, S7A-B), a phenomenon also seen in mouse hotspots (38). Hotspots of the human A-allele analysed here overlap exons in 3,486 genes. Therefore, we sought to assess health impacts of mutations arising from meiotic breaks through gnomAD and ClinVar, a large-scale database of polymorphisms with evidence regarding their clinical significance (52). First, we examined how often these mutations lead to disruption of genes in the population through gnomAD. These data exclude individuals with severe paediatric disease and their first-degree relatives and may represent an underestimate of the overall impact of mutations. To provide a complementary viewpoint, we assessed pathogenic variation in ClinVar.

The number of gnomAD predicted loss of function (pLOF) mutations in hotspots is 75% (95% CI=[71%, 80%]) higher for indels and 67% (95% CI=[63%, 71%]) higher for single-base substitutions than expected from the proportion of DNA sequence in hotspots (41). To assess whether this is driven solely by the increased mutation rates established above, we compared the fraction of mutations that lead to pLOF in hotspots with that in the genome. We found that a single-base substitution in a hotspot is 38% (95% CI=[35%, 42%]) more likely to be pLOF than one elsewhere. For indels, the corresponding value is 28% (95% CI=[25%, 31%]). These impacts are consistent with the over-representation of hotspots near exons (41).

For a mutation, we can estimate the probability that it arose from meiotic break repair based on its distance from a hotspot centre and the over-representation of that variant type in hotspots (Fig. S7C-D). Here, we report pLOF mutations that are most likely to have arisen as a result of programmed breaks (average p=0.80, see Table S2 for additional variants).

We observed 206 SVs and 77 indels disrupting 278 genes that meet these criteria. Amongst them, 40 genes have multiple pLOF variants attributable to meiotic DSBs (31 SVs impact more than one gene) (Table S2). These genes are linked with a range of X-linked and autosomal disorders, e.g., *TMLHE* (X-linked Autism), *CDKL5* (Developmental Encephalopathy), *FANCD2* (Fanconi anaemia), *FLT4* (congenital heart defects), *FTCD* (Glutamate formiminotransferase deficiency), and *DOCK8* (DOCK8 immunodeficiency syndrome) (Fig. 5B, S7E, Table S2). Amongst these 278 genes, to the best of our knowledge, mutations in only *SHOX*, the *VCX* gene family, and *PRDM9* itself have previously been attributed to meiotic recombination (24, 36, 53, 54).

Genes in ClinVar have received variable degrees of investigation. To prevent confounding for this reason or biological factors such as a difference in tolerance for mutations, we took a stringent approach: we compared pathogenic polymorphisms in exons that overlap hotspots with other exons of the same gene (41). Specifically, we investigated multi-exonic genes with at least one pathogenic exonic polymorphism (n=1,298). We found that hotspot-overlapping exonic regions contain 41% more pathogenic mutations than non-overlapping ones on average (95% CI=[11%, 80%], p=5x10^-4^ (41)). The impact on exonic regions closer to hotspot centres is even higher (with nearly double the rate of pathogenic mutations ±100bp from hotspot centres (41)).

We identified 81 genes that have hotspot-overlapping exons with statistically significant increases in pathogenic mutations after Bonferroni-correction for multiple testing (Fig. 5C, Table S3). These genes include HEXA (Tay-Sachs disease), CDKN1B (Neoplasia), GATA1 (Thrombocytopenia, Thalassemia), and SH2D1A (Lymphoproliferative syndrome).

Collectively, these data establish that meiotic breaks are a previously under-recognised cause of human disease.

## Footprints of single-base substitutions implicate translesion DNA polymerases in meiotic break repair

Many exogenous and endogenous factors that impact DNA have characteristic ‘mutational signatures’. These signatures have proved powerful in understanding the molecular processes driving many cancers (55). For example, the trinucleotide context of mutated bases, i.e., the bases immediately upstream and downstream of the mutated base can help distinguish their underlying causes.

**Figure 6:**
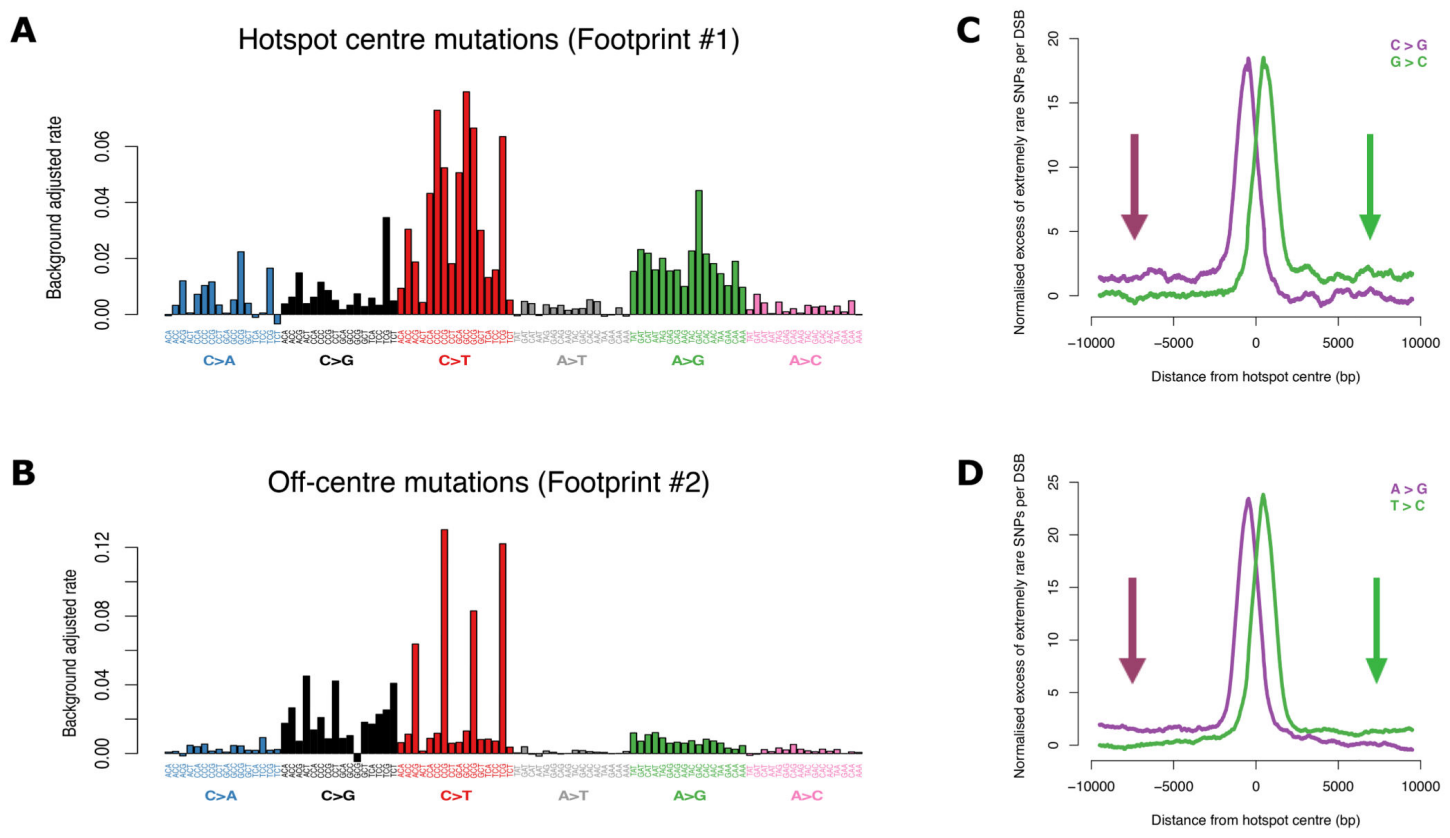
Mutational signatures of single-base substitutions in hotspots. **(A)** Trinucleotide mutational signature showing the number of mutations observed per base in the central region of autosomal hotspots (± 50 bp from the centre of the PRDM9 binding motif) after correcting for the local background rate. The rates shown include the reverse complement. A positive value implies a higher rate inside hotspots than background and vice versa. The figure corrects for differences in sequence composition in and around hotspots. **(B)** As (A) but for the off-centre peaks in X chromosome hotspots. The trinucleotide context of C>G changes resembles the preferences of the AID/APOBEC family, particularly AID, APOBEC3F, and APOBEC3G (58, 59). APOBEC3F is strongly expressed in human testes (59). **(C)** Fold excess in the number of extremely rare C>G and G>C mutation per ‘C’ and ‘G’ base per DSB, respectively, in and around autosomal hotspots (n=25,440; 1 kb moving window). The figure corrects for variation in sequence composition. Arrows highlight the long-range excess of strand-asymmetric mutations away from hotspot centres. **(D)** As (C) but for A>G and T>C mutations.

We therefore assessed the trinucleotide mutational signature of the central and off-centre peaks of single-base substitutions identified above (Footprints #1 and #2, Fig. 2B,G, respectively). We observed significant variability in mutation rates depending on the trinucleotide context in both footprints (Fig. 6A-B, S8A-B). In additional to single-base substitutions, the central peak mutational signature includes 1 bp indels (Fig. 3B). None of the known mutational signatures inferred from cancer genomes (55) is a good fit for this signature. One possible mechanism is a non-canonical pathway for processing meiotic breaks that could enable repair via end-joining (Fig. S8C) (56, 57).

In the off-centre peaks, which reflect the regions typically outside the D-loop (Fig. 1A), the trinucleotide context of C>G mutations (Fig. 6B) is consistent with preferences of AID/APOBEC cytosine deaminases, which are known DNA mutators (58, 59). C>T mutations in these peaks are strongly elevated in a CpG context (Fig. 6B), consistent with both spontaneous and enzymatic deamination in ssDNA (49). These data indicate that DNA outside the D-loop accrues more cytosine deamination than DNA within it, which is subsequently repaired incorrectly.

Translesion synthesis (TLS) DNA polymerases, e.g., Rev1, Polη, PolϚ, are strong candidates for effecting this repair (Fig. S9). TLS can lead to C>G, C>T, and A>G mutations (19, 49, 60–62), whilst a non-exclusive possibility for C>T mutations is replication following cytosine deamination (49). C>G mutations are a telltale signature of REV1 (49) and repair by mismatch-repair machinery leveraging Polη can give rise to A>G mutations (63). Our analysis of published single-cell RNA-seq data from mouse testes (64) shows that several TLS polymerases are highly expressed at the relevant timeframe in meiosis (Fig. S9A-F). TLS involvement in yeast meiosis is indicated by two-hybrid associations (65) and they mediate cell-cycle dependent repair stimulated by RAD51 in somatic cells (66–68). Our findings thus suggest that TLS polymerases, potentially mediated by RAD51 (Fig. 2F), are involved in filling the gap that remains in resected DNA distal to the break-site after HR. A non-exclusive possibility is reduced efficiency of mismatch repair in the region outside the D-loop (49).

In addition to the off-centre peaks, C>G and A>G mutations exhibit long-range (>10kb) strand-asymmetric mutations (Fig. 6C-D). This signature is consistent with TLS in the context of break-induced replication (BIR), a distinct and highly error-prone repair pathway that generates long tracts of ssDNA via a migrating D-loop (Fig. 1A)(19)(69).

## Sequence features of indels implicate template-switching and microhomology-mediated end-joining in meiotic break repair

**Figure 7:**
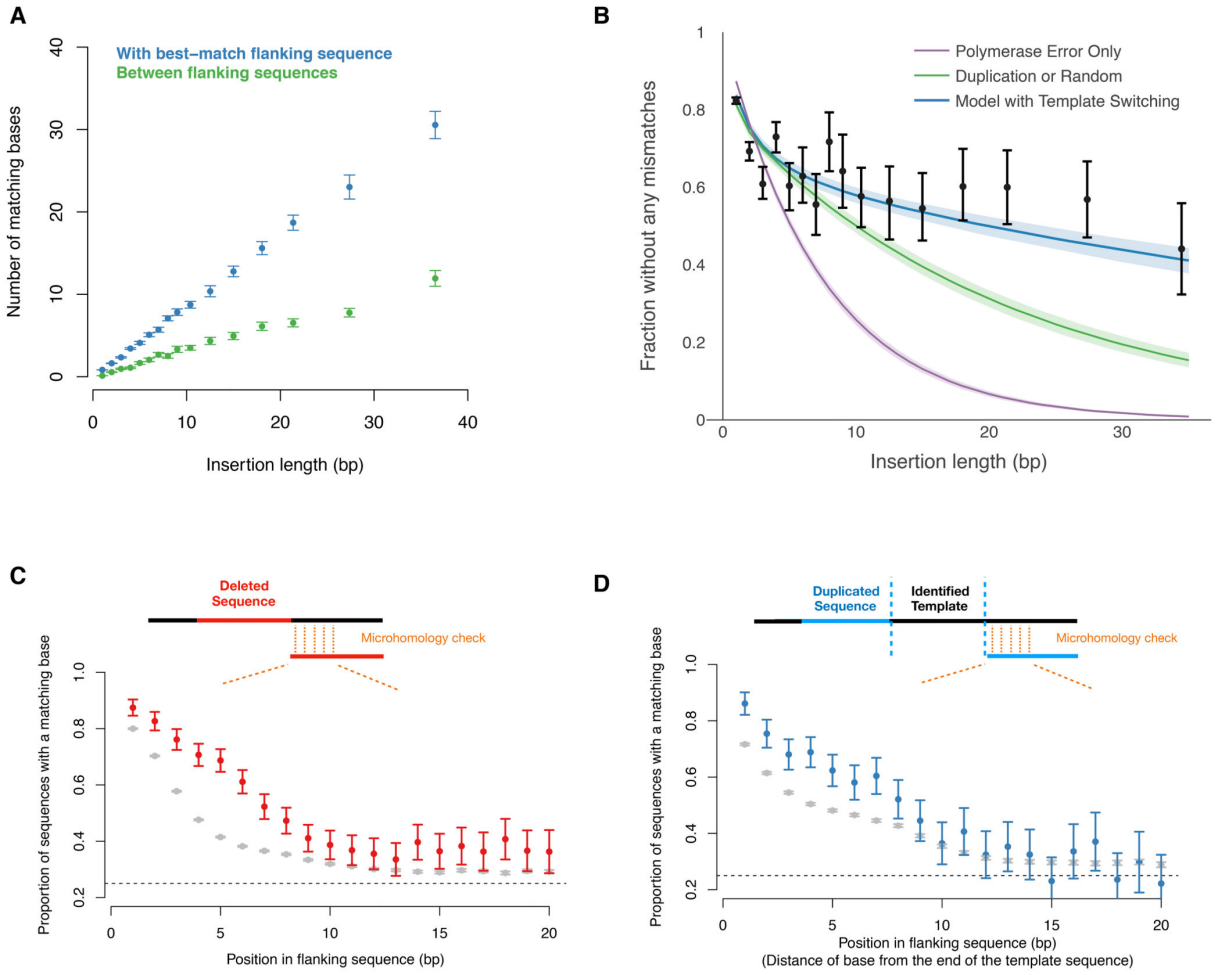
Mechanisms underlying insertions and deletions in hotspots. **(A)** Sequence homology between the inserted sequence and the more similar of its two flanking sequences (blue) for insertions with both breakpoints in unique DNA (n=12,029) (mean=0.82). The homology between the flanking sequences themselves (green) is shown as control. Error bars show two standard errors for the estimate of the mean. **(B)** The proportion of inserted sequences that are a perfect match to their best-match flanking sequence (y-axis) are shown relative to the insertion length (x-axis). We fitted and tested three models for generating mismatches: random polymerase errors (purple, ‘Polymerase error only’), copying the adjacent sequence or a random sequence from the genome, with polymerase errors (green, ‘Duplication or random’), copying the adjacent sequence or a sequence with varying degrees of homology with it, with polymerase errors (blue, ‘Model with template switching’). The distribution of template homology and probability of polymerase errors were inferred from the data under this model (See also Fig. S10). **(C)** The proportion of deleted sequences wherein the base at a given position matches the base at the corresponding position in the flanking DNA sequence for deletions arising from meiotic breaks (red) relative to those in local background (grey). The average microhomology across sequences inside a hotspot is the weighted mean of microhomology due to background processes and that due to meiotic break repair. We infer the meiosis-specific signal by subtracting out the background signal. Error bars show two standard errors of the mean. Sequences >=5bp in length with a breakpoint within the PRDM9 binding motif were included. **(D)** As (C) but for duplications, which are compared with the DNA sequence flanking the template. Insertions that were not perfect duplications were excluded due to uncertainty in identifying the correct position for comparison.

To understand the mechanisms generating short insertions, we compared each inserted sequence in unique DNA with its flanking sequences. We observed that 82% of bases matched, on average, between the inserted sequence and the more similar of its right- and left-flanking sequences, which is not expected by chance (Fig. 7A, p<2x10^-16^). The canonical model for generating insertions is “polymerase slippage” (27, 70). Under this model, the polymerase performing DNA synthesis for break repair disassociates with its template and subsequently re-attaches to a segment already synthesized, which leads to a duplication. Mismatches from the template (i.e., an insertion that is non-identical) could be due to random polymerase errors or by copying from an incorrect template (19). However, neither random polymerase errors nor copying from a random template are able to explain the observed data, alone or in combination, especially for insertions longer than ~10 bp (Fig. 7B, S10A-C) (41).

We reasoned that the pattern of mismatches could be explained if some insertions are generated by successively copying from multiple templates such as the correct and one or more incorrect templates (41), a phenomenon known as “template-switching” (19). To capture this, we modelled insertions as arising from templates with varying degrees of similarity with the correct template, while also allowing for random polymerase errors. We used Markov chain Monte Carlo (MCMC) to sample template properties and polymerase error rates consistent with the data and found that this model captures the observed distribution of mismatches (Fig. 7B, S10A-D). Under this model, we inferred that 63% (95% CI=[60%-66%]) of insertions are generated by copying the same template more than once (i.e., side-by-side duplications) (Fig. S10E) with a polymerase error rate of 1.2% (95% CI=[1.0%-1.5%]) (Fig. S10F), which is consistent with properties of TLS polymerases (68, 71). The remaining 37% of insertions are combinations of homologous and non-homologous sequence, consistent with template-switching (Fig. S10E). A tendency to fall off their template sequence after incorporating only a small number of nucleotides, i.e., low processivity, is another hallmark of TLS polymerases (68).

End-joining repair pathways that ligate DNA on either side of the break can lead to deletions (72). Although these pathways are thought to be suppressed in meiosis, we hypothesized that end-joining is used as a backup mechanism for sites that remain partially or entirely unrepaired at a critical stage (late pachytene). Work in mitotic cells, HR-deficient cancers, and in vivo in worm provide evidence that the microhomology-mediated end-joining pathway mediated by DNA Polymerase θ (also known as theta-mediated end-joining or TMEJ for short) can repair resected break-sites previously occupied by HR proteins (72–74). In meiosis, a switch from HR to TMEJ at unrepaired programmed DSB sites would lead to germline mutations. Note that, in addition to deletions, this pathway can generate insertions, e.g., when DNA next to the break-site has already been extended, as analysed above (Fig. 7A-B). Accordingly, we checked whether sequences at deletion and insertion breakpoints show evidence for microhomology (we restricted the analysis of insertions to side-by-side duplications since the template can be identified in those cases).

For autosomal indels that have both breakpoints in unique DNA, we examined the inserted and deleted sequences for microhomology. The vast majority of indels in hotspots showed significant microhomology at the breakpoint, which is not expected by chance (Fig. S11A; p<2x10^-16^).

Since other end-joining pathways can also exhibit some microhomology (usually between 0-3 bp for non-homologous end-joining or NHEJ for short) (reviewed in (72, 75)), we compared the properties of hotspot indels with those in the local background. Indels in hotspots exhibit significantly greater microhomology than those outside (Fig. 7C-D). Microhomologies in insertions and deletions in hotspots are similar to each other (Fig. S11B-C), which is consistent with them arising from a shared aetiology. Microhomologies range mainly between 1-10 bp but are sometimes higher, consistent with known properties of TMEJ (72, 76).

A model that is consistent with these data allows for deletions to arise through TMEJ between resected sites flanking DSBs and insertions via TMEJ between sites where some DNA re-synthesis has already taken place (Fig. S11D). Polθ, which is absent in yeast, is expressed in only a few tissues in human and mouse, with highest expression in testis (77). Furthermore, our analysis of single-cell RNA-seq data from mouse testes (64) shows that Polθ and Lig3, the major ligase mediating TMEJ (72), are highly expressed during the relevant timeframe in meiosis, i.e., pachytene (Fig. S11E-F). Collectively, these data strongly suggest that TMEJ is a major mutagenic force in the human germline.

## Provenance of structural variants in hotspots

The canonical model for SVs associated with the recombination machinery is non-allelic homologous recombination (NAHR), which posits that SVs are generated through ectopic pairing between large DNA segments with high sequence similarity (28). Although a good fit for several mutations that underlie specific genomic disorders, NAHR cannot explain the overall pattern of SVs that we have observed (Fig. 3C, S12A). The NAHR model predicts an excess of deletions over insertions (specifically duplications) (28). In contrast, we have shown a strong excess of insertions over deletions in autosomal hotspots (Fig. 3C, S12A).

**Fig 8:**
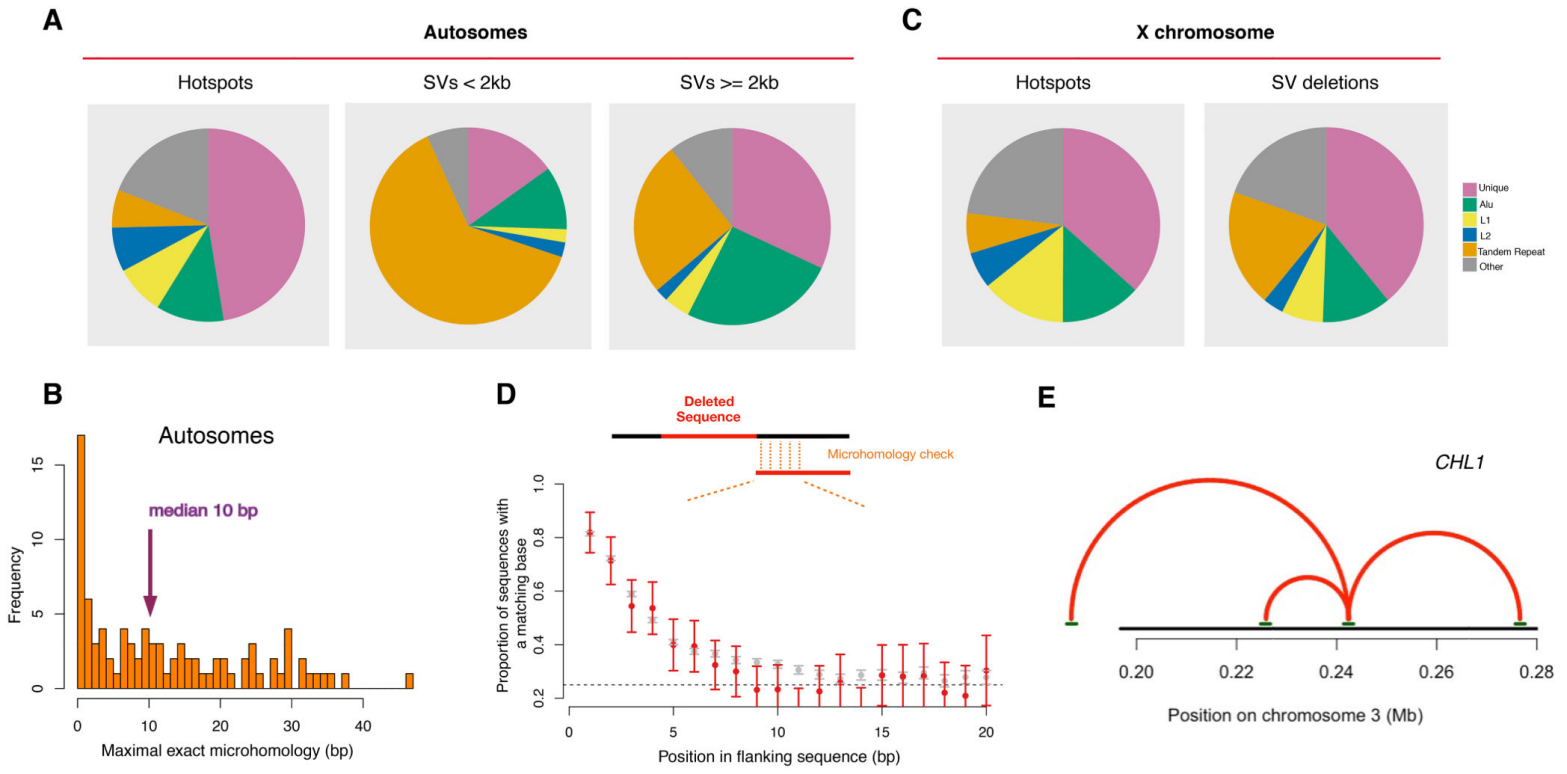
Provenance of structural variants in hotspots. **(A)** Sequence context of autosomal hotspot centres (left) and hotspot-proximal SV breakpoints in deCODE-SV for SVs smaller than 2 kb (middle) and 2 kb or larger (right). The contexts/repeat families shown are Unique DNA (pink), Alu (green), L1 (yellow), L2 (blue), Tandem Repeat (orange), and others (grey). SVs with breakpoints within 100 bp of hotspot centres were included. **(B)** Histogram of the maximal error-free microhomology between the deleted sequence and its flanking sequence for SV deletions smaller than 2kb that had neither breakpoint in a tandem repeat sequence (n=106) and had a breakpoint within 100 bp of a hotspot centre. Complex events (i.e., deletions with accompanying insertions, n=16) were excluded due to uncertainty is identifying the correct position for comparison. **(C)** As (A) but showing the context of X chromosome hotspot motif centres (left) and hotspot-proximal breakpoints (right) for SV deletions (all sizes). (D) As Fig. 7C but for the X chromosome **(E)** A sub-telomeric locus with SV deletions (red arcs) between hotspot centres (within 200 bp from the PRDM9 motif midpoint). Hotspots (horizontal green lines) and the *CHL1* gene body (black line) are shown.

Detailed sequence and breakpoint analysis of several complex SVs in autosomal hotspots revealed features observed in short indels above, specifically template-switching and signatures of TMEJ (Fig. S13). This suggests that mechanisms underlying short indels, i.e., annealing of extended or unextended ssDNA flanking the break-site, may also explain many SVs. If this hypothesis is correct, we would expect an excess of SVs in a size range within the extent of resection (~2kb). Comparison of SV sizes confirms that this is the case for both insertions and deletions (p=5x10^-22^ for deletions, p=10^-32^ for insertions, Fig. S3B, S12B-G) (41). The preponderance of SVs smaller than 2kb with breakpoints in TRs (Fig. 8A) is consistent with TMEJ. Amongst non-TR SV deletions and duplications smaller than 2kb, microhomology was observed in 84% and 94% of events respectively, with median microhomologies of 10bp and 15 bp respectively (Fig. 8B, Fig. S12H) (41). We consider mechanisms underlying the small proportion of hotspot SVs that are larger than 2kb in (41) (Fig. S12I-J).

Finally, we examine the abundance of SV deletions in X chromosome hotspots (Fig. 3D). In addition to being more numerous per base pair, they are systematically larger: their average size (5.4kb) is twice those in autosomal hotspots (2.7kb), with 34% being larger than 2kb (compared with 12% on the autosomes) (p=2x10^-7^) (Fig. S12K). While the number of SV deletions is correlated with hotspot intensity and background mutation rate on both the X chromosome and the autosomes, their relative impacts are distinct: hotspot intensity is a stronger predictor on the X while background mutation rate is a stronger predictor on the autosomes (41). Consistent with this, the proportion of SV deletions in unique DNA in X chromosome hotspots resembles the proportion of hotspots themselves (Fig. 8C). The median microhomology in non-TR SV deletions in X chromosome hotspots is 1 bp, which is lower than that on the autosomes (p=2x10^-4^) and similar to expectations from NHEJ. Microhomology in short deletions in X chromosome hotspots is also lower than autosomal hotspots and similar to deletions outside hotspots (Fig. 8D).

Collectively, these analyses suggest reduced use of microhomology-mediated repair on the X and higher impact of a process such as NHEJ, which directly ligates DNA ends. Why might X chromosome breaks be repaired differently from those on the autosomes? Note that the X chromosome lacks a homolog in male meiosis and regulation and repair of breaks thereon differ from the autosomes in several respects (78, 79). Whilst the extensive DNA resection that accompanies meiotic breaks is expected to disfavour NHEJ (75), it is possible that some X chromosome breaks are processed without DNA resection, for example, through the alternative break-end processing mechanism discussed above (Fig. S8C) (11, 56, 57). Another possibility is that resected DNA is filled-in prior to NHEJ (80, 81). The X chromosome SVs we observe could be due to NHEJ between sites of programmed breaks or between sites of programmed and sporadic breaks. The first of these possibilities can be tested with the present data and, although uncommon, we find significant evidence for end-joining between hotspot centres (p=0.009, polarisation test (41)). These events are predominantly near telomeres (Fig. 8E, 9/11 are within 2 Mb of chromosome ends, p=4x10^-15^) and are over-represented on the X chromosome (3/11, p=0.008). Sub-telomeric hotspots have an intense burden of programmed breaks in male meiosis and exhibit distinct kinetics of repair (82, 83). Our analyses suggest that, as with the X chromosome, they may rely on otherwise disfavoured pathways to repair some of them.

## Discussion

Induction and repair of hundreds of programmed DNA double-strand breaks is a central part of the creation of eggs and sperm. Despite their multi-generational impacts on human health and diversity, an understanding of errors in these processes has been hampered because they are individually very rare.

Here, we have shown that they are collectively common: 1 in 4 sperm and 1 in 12 eggs has a de novo mutation specifically due to meiotic breaks. We demonstrate that the previously reported link between de novo single-base substitutions and crossovers is only the tip of the iceberg, with the overall burden due to meiotic breaks being almost an order of magnitude higher. These data further show that the mutation rate per break is ~3-fold higher in paternal relative to maternal meiosis. This is comparable to the genome-wide difference in the number of DNMs inherited from fathers relative to mothers. Recent work provides evidence that the higher mutation rate in males is driven primarily by differences in the balance between DNA damage and repair in males and females (84). If the lower accuracy of repair that we have observed for meiotic breaks in males holds across germline breaks more generally, it could explain the higher rate of paternally-inherited mutations genome-wide.

In addition to single-base substitutions, we find that DSBs lead to mutation rates 100-1300 times higher per break for indels and SVs than would be expected in those regions in the absence of a break. These rates are impacted by the size, nature, and context of the mutations, with SVs biased towards insertions in the autosomes and deletions on the X chromosome. These findings are consistent with previous work in humans where the latter exists, namely, excess of DNMs near crossovers (7), excess of rare SNPs in hotspots (20, 23), and tandem repeat instability (24, 25). Despite fewer than 1% of potential hotspot sites undergoing a break in any given meiosis, the high mutation rates per break make hotspots a significant force in germline mutagenesis.

We provide multiple lines of evidence that a repertoire of error-prone DNA repair mechanisms, e.g., translesion synthesis, microhomology-mediated end-joining, break-induced replication and non-homologous end-joining are involved in human meiotic break repair. We find that, for single-base substitutions, distinct mechanisms are at play at the hotspot center, and inside and outside the D-loop. The vast majority of autosomal indels and SVs show evidence for microhomology-mediated end-joining. In contrast, our analyses show that the canonical model for generating SVs, i.e., non-allelic homologous recombination, cannot explain them. It is surprising that many of these pathways, which are normally associated with repair in somatic cells, especially cancer cells, and mutant organisms (11, 18, 19, 48, 49, 56, 57, 60, 65, 69, 72, 73), are active in response to programmed breaks in human germlines at large.

Comparison between the autosomes and the X chromosome suggests that, whilst microhomology-mediated end-joining is mutagenic, it protects against larger and potentially even more deleterious mutations. The mutation burden is particularly high near telomeres and on the X chromosome, both of which face specific challenges in male meiosis (78, 82). Collectively, these data suggest that many meiotic mutations accrue at times of stress, e.g., when breaks cannot be repaired with the intended pathway or within the requisite timeframe.

Mis-repair of meiotic breaks is thereby a cause of disease, with 41% increase, on average, in the number of pathogenic mutations in exonic regions overlapping hotspots genome-wide. Our analyses have identified 81 genes with significantly higher pathogenic mutations due to this process. Furthermore, we have identified 278 genes with loss-of-function mutations attributable to meiotic breaks (5 genes overlapped in these lists). These 354 genes are involved in a range of developmental disorders and cancers and, to the best of our knowledge, only 3 of them have previously been linked with mutations generated as a consequence of meiotic break repair.

Sexual reproduction requires homologous chromosomes to pair up in order to re-segregate into haploid gametes. Adaptation of the tools of DNA repair to achieve this challenging task lies at the heart of meiosis and multiple aspects of this process are conserved from yeast to human (1, 85). The meiotic program must thus find an equilibrium between the risk of infertility due to insufficient breaks (86) and the cost of pathogenic mutations. Our analyses show that this cost is considerably more severe than had been suspected.

The over-representation of programmed breaks near exons in humans and at transcription-start sites in many species is therefore surprising and suggests that there is an evolutionary benefit to positioning breaks near genes. It is possible that the chromatin environment in these regions promotes repair, thereby increasing the likelihood of successful chromosome pairing (87). If this were to be the case, it would imply that the increase in fertility afforded by this strategy outweighs the burden of genetic disease from mis-repair of DSBs.

The evolutionary cost of incorrectly repaired breaks is predicted to be particularly acute for the sex chromosomes, where lower effective population sizes and reduced crossing over imply that efficiency of natural selection will be lower. Concentration of breaks towards telomeres in males and lower gene density on the sex chromosomes may, in part, reflect an evolutionary response to the mutation burden of DSBs. Extensive DNA resection accompanying breaks, while incurring a clear mutational cost, likely contributes to correct chromosome pairing and safeguards against more catastrophic genome instability. The mechanisms underlying meiotic recombination thus perform a delicate evolutionary balancing act between the benefit of sexual reproduction and the burden of genetic disease.

## Materials and Methods

### Hotspot calling

We used ChIP-seq data for single-stranded DNA bound to DMC1, which was measured in testes of a human male homozygous for the A-allele and one heterozygous for the C and L4 alleles (20, 39). Hotspot were called and their DMC1 intensities were estimated using our peak-calling methodology (38). We identified the most likely PRDM9 binding site within each hotspot using a motif calling algorithm (88) and defined its midpoint to be the hotspot centre. One AA hotspot (out of 28,286), whose intensity estimate was a large outlier, was excluded from analyses involving hotspot intensities.

### Estimating the full burden of de novo single-base substitutions in human recombination hotspots

We used published data of de novo mutations (DNMs) and crossovers identified in 2,976 Icelandic trios (7). Only a subset of programmed meiotic breaks are repaired with a crossover. Detailed methodology for inferring the burden of DNMs due to meiotic breaks, including those not associated with a crossover, is provided in (41).

### Footprints and rates of single-base substitutions, indels, and structural variants in hotspots

We used the Gnomad-v3.0 dataset (45) and only used variants that passed all gnomAD filters. In addition, we restricted to variants that had a positive variant quality score (AS_VQSLOD > 0). This included 368 million SNPs and 64 million indel calls. We then filtered for variants with allele frequency <10^-3^ as extremely rare SNPs are recent enough for the impact of selection and meiotic drive to be small and have proven to be a powerful source for research in human mutation (23). This provided 341 million SNPs and 56 million indels.

In the plots for base-specific single-base substitutions, we corrected for differences in sequence composition with a base-by-base normalisation, e.g., in the case of C>T mutations we divided the number of extremely rare C>T SNPs at each position (relative to the hotspot centre) with the number of times a C base was observed at that position in the reference genome. The same approach was extended to mutations within each trinucleotide context. Some sites may have experienced more than one independent mutation in the genealogical history of the individuals in the gnomAD sample set (89). Since each site is reported only once per mutation type in gnomAD, it is possible that we underestimate the mutation burden for the most strongly enriched mutation types, e.g., CpG>TpG in the off-centre peaks.

We used SV calls from the Icelandic population (deCODE-SV) (24) and the gnomAD populations (gnomAD-SV version 2.1) (50).

We inferred the per-DSB fold excess in mutations as follows. Consider a hotspot and let *B* be the event of a break in it in a meiosis and *M* be the event that it incurs a mutation at a specific position in that meiosis.

P(M)=P(M|B)P(B)+P(M|B′)P(B′)

Let the background rate *P*(*M*|*B*′) be *r_bg_*. Since *P*(*B*) ≪ 1,

$$P\left(M|B\right)\approx \left(P\left(M\right)-r_{bg}\right)/P\left(B\right)$$

We wish to calculate the mutation rate per break averaged across *n* hotspots and *m* meiosis in the sample, i.e.,

$$\frac{1}{mn}\sum_{i=1}^{m}\sum_{j=1}^{n}P\left(M_{ij}|B_{ij}\right)=\frac{1}{mn}\sum_{i=1}^{m}\sum_{j=1}^{n}\frac{P\left(M_{ij}\right)-r_{bg_{ij}}}{P\left(B_{ij}\right)}$$

The probability of a break in a hotspot in individual meioses in the genealogical history of a sample (*P*(*B_ij_*)) is not known. However, we have estimated the average probability of a break in an A-allele hotspot from a present-day Icelandic sample (0.8907%), as described in (41). Assuming that $P\left(B_{ij}\right)\approx \mu_{B}=\frac{1}{mn}\sum_{i=1}^{m}\sum_{j=1}^{n}P\left(B_{ij}\right)$,

$$\frac{1}{mn}\sum_{i=1}^{m}\sum_{j=1}^{n}P\left(M_{ij}|B_{ij}\right)\approx \frac{\frac{1}{mn}\sum_{i=1}^{m}\sum_{j=1}^{n}P\left(M_{ij}\right)-\frac{1}{mn}\sum_{i=1}^{m}\sum_{j=1}^{n}r_{bg_{ij}}}{\mu_{B}}$$

We calculated the background rate of mutations as the number of mutations per base in the regions 5 kb - 10 kb from hotspot centres, excluding regions that overlap with another nearby hotspot. The number of meioses in the sample is unknown, therefore we are able to infer only the fold-excess, which is reported.

$$\frac{\frac{1}{mn}\sum_{i=1}^{m}\sum_{j=1}^{n}P\left(M_{ij}|B_{ij}\right)}{\frac{1}{mn}\sum_{i=1}^{m}\sum_{j=1}^{n}r_{bg_{ij}}}\approx \frac{1}{0.008907}\left(\frac{\sum_{i=1}^{m}\sum_{j=1}^{n}P\left(M_{ij}\right)}{\sum_{i=1}^{m}\sum_{j=1}^{n}r_{bg_{ij}}}-1\right)$$

Note that this estimate is conservative for gnomAD because a significant proportion of populations included in the gnomAD dataset have PRDM9 alleles with binding properties distinct from those of the A-alelle.

In the plots of the per-DSB excess in indel and SV mutation rates, we have used both breakpoints unless otherwise specified. In specific plots, the hotspot-proximal and the hotspot distal breakpoints were identified by comparing their respective distances to the closest hotspot centre and shown separately.

To calculate point estimates of the elevation of mutations per break in hotspots, we used counts of indels and SV breakpoints in the central 100 bp of hotspots, since DNA double-strand breaks are concentrated mainly in this region in mouse hotspots (46). 95% confidence intervals were estimated using bootstrap (10,000 bootstrap samples for SVs and 2,000 for indels).

### Repetitive DNA

The repeat context of indel breakpoints was identified using the RepeatMasker track for Build 38 downloaded from the UCSC Genome Browser at https://genome.ucsc.edu/cgi-bin/hgTables.

Tandem Repeat (TR) annotations were downloaded using the simpleRepeat track from the UCSC Genome Browser, which is based on Tandem Repeat Finder (TRF) (90). Tandem repeats are defined as “two or more adjacent, approximate copies of a pattern of nucleotides” (https://tandem.bu.edu/trf/trf.html) and include microsatellites, minisatellites and other tandem repeats with a period size between 1 bp and 2 kb. In cases where the RepeatMasker and simpleRepeat track both annotated the same sequence, we used the Tandem Repeat annotation.

### Modelling insertions

Only indels with both breakpoints in the same context were used in context-specific analyses. Indels for which neither breakpoint overlapped a RepeatMaster or Tandem Repeat sequence were deemed to be in “unique DNA”.

For specific analyses measuring indel homology and microhomology in unique DNA (Fig. 7), we performed more stringent filtering to avoid over-estimating (micro)homology from indels in repetitive DNA that may have escaped the filters above. In the event of multiple equivalent insertion or deletion positions, as is the case for many indels in TRs and homopolymer runs, the first of those positions is reported in gnomAD. Therefore, we filtered out any sites with more than one insertion (or deletion) in these analyses as a further check against inclusion of homopolymer or tandem repeat sequences.

For perfect side-by-side duplications, it is not possible to determine the true breakpoint. For a duplication of size *n*, the true insertion point can be any one of *n+1* possible sites: immediately upstream or downstream of the duplicated sequence or anywhere in between. The first of these positions is reported in gnomAD, as mentioned above. We also use this representation, without loss of generality. Imperfect duplications can also have multiple representations. In such cases, for consistency, we chose the representation that maximized homology with the right-flanking sequence, which was almost always the one reported in gnomAD (98%). We excluded insertions where the findings (of, say, microhomology) were different for these different representations to avoid biasing the results. This was also frequently the case in complex SVs where the inserted sequence showed homology with more than one locus.

We modelled the provenance of insertions using Markov Chain Monte Carlo (MCMC). Detailed methodology for inferring the provenance of insertions is provided in (41).

### Gene annotations

Gene and exon annotations were downloaded from Gencode (v42). We restricted to protein coding genes and used Ensembl canonical transcripts to define gene and exon boundaries.

### Estimating the probability that a variant emerged as a consequence of meiotic break repair

Consider the fold-enrichment *f* in the number of SV and indel breakpoints at a given distance *d* from the hotspot centre. We infer that the probability that a variant, which has its hotspot-proximal breakpoint at this distance, arose due to recombination is *(f-1)/f*. We calculated this value for each base pair distance *d* from hotspot centres on average (Fig. S7C-D). We assumed that *f* decreases monotonically with distance from the hotspot centre (this assumption is well supported by the data, see Fig. 3) but that the data is noisy. Therefore, we fitted a piecewise-constant monotonic function to the data with a node point at every *d*. The sum of squares of the deviation between the data and regression function was minimised, which is equivalent to maximum likelihood estimation under an assumption that errors are normally distributed. The resultant quadratic programme was solved in R using the quadprog package.

### ClinVar data

We downloaded the ClinVar data on January 19, 2023. We restricted to variants that had the ClinSigSimple field set to 1, flagging pathogenic and likely pathogenic variants.

## Supplementary Material

Data S1

Supplementary material

Table S2

Table S3

## Data Availability

DMC1 hotspots identified in this study are provided in the supplementary materials. Computer code is available at (91). The human variation datasets used are publicly available and cited in the paper.

## One-Sentence Summary

A surprisingly large number of germline mutations in humans stem from programmed DNA double strand breaks induced by the meiotic recombination machinery, which are repaired via a repertoire of unexpected mechanisms and lead to a range of autosomal and X-linked diseases.
