# Supplementary material for: Meiotic DNA breaks drive multifaceted mutagenesis in the human germline
Source: Science. Author manuscript; Available in PMC 2023 Dec 9. (PMC7615360; doi:10.1126/science.adh2531)
Supplement: Supplementary material [file EMS191910-supplement-Supplementary_material.pdf]

Science
AAAS

Supplementary Materials for

Meiotic DNA breaks drive multifaceted mutagenesis in the human germline

Authors: Robert Hinch, Peter Donnelly, Anjali Gupta Hinch*

*Corresponding author. Email: anjali.hinch@well.ox.ac.uk

The PDF file includes:

Extended Methods
Supplementary Text
Figs. S1 to S13
Table S1
Captions for Tables S2 and S3
Caption for Data S1
References (*92-98*)

Other Supplementary Materials for this manuscript include the following:

Tables S2 and S3
Data S1

Extended Methods

Estimating the burden of de novo point mutations in human recombination hotspots

We applied our hotspot calling approach to published data measuring binding of the meiosis-specific protein DMC1 in testes of a human male homozygous for the A-allele and one heterozygous for the C and L4 alleles (*20*, *39*). Note that measures of DMC1 in hotspots reflect the number of DSBs and the time it takes to repair them (*9*, *83*). We refer to these hotspots as AA and CL4 hotspots henceforth. We identified the most likely PRDM9 binding site within each hotspot (*88*) and defined its midpoint to be the hotspot centre. Different processes occur at different scales within hotspots (Fig. 1A). Nevertheless, for simplicity and clarity, we assume a size of 2 kb unless noted otherwise.

We compared our AA hotspot set with crossovers identified in 2,976 Icelandic trios (*7*). The hotspot set overlapped 81% of male and 66% of female crossovers and represented an improvement of 50% in specificity and 20% in sensitivity of hotspot calling over a previously published hotspot set using the same underlying ChIP-seq data (*20*). Although we have shown it for illustrating the concepts in Fig. 1B, our analyses for calculating the mutation burden do not depend on hotspot intensity. Nevertheless, for completeness, we have repeated the analyses for females with another independent hotspot set (see details below), and found that it provides consistent estimates.

DNA sequence inside hotspots is subject to many factors generating mutations, just as DNA outside hotspots is. Some factors impacting mutation rates (e.g., replication timing and GC-content) vary between genomic regions, therefore it is important to account for local mutation rates. We do so by calculating the DNM rate in the immediate vicinity of each hotspot (between 5kb and 20kb from hotspot centres). To check that DNM rates are not higher a priori in recombination hotspots, we compared DNM rates in Icelandic trios in AA and CL4 hotspots. PRDM9 alleles with binding properties similar to the A-allele (referred to as A-like alleles) comprise the vast majority (~95%) of PRDM9 alleles in northern Europeans (*37*, *39*, *92*). In contrast, C-like alleles (which include the L4 allele (*39*)) are rare in European populations (*39*, *92*) and bind a distinct set of hotspots (*37*, *39*, *92*). We observed that the local background DNM rate is in line with the DNM rate in C-like hotspots in the Icelandic population (Fig. S1A). We conclude that the DNM rate inside hotspots in the absence of recombination is similar to the rate outside them. Note also that our approach is conservative: a subset of the mutations, which we attribute to background processes, may be long-range mutations (e.g., Fig. 6C-D, clustered mutations in older mothers (*7*)).

To calculate the number of DNMs that are due specifically to the recombination machinery in each parent, we corrected for (i) local mutation rates and (ii) systematic differences in power to identify parent-of-origin of DNMs (Fig. S1A-B). We counted the total number of paternal and maternal DNMs within 1.5 kb of hotspot centres and the background regions (5-20 kb) as discussed above. For a subset of DNMs, the parent-of-origin was available (*7*). We calculated the power to identify the parent-of-origin in those cases by dividing the number of DNMs for which parent-of-origin was identified by the total number of DNMs in that set. We then

calculated the expected number of DNMs by dividing the observed number of DNMs by the power to infer parent-of-origin. The number of DNMs due to the recombination machinery was inferred to be the total expected number of DNMs subtracted by the background expected number of DNMs. We also counted, for each sex, the number of DNMs inside hotspots that were or were not associated with a crossover. Note that the power to infer parent-of-origin was inferred separately for each of these cases (i.e., DNMs with crossover in hotspots, DNMs without crossover in hotspots, background DNMs). 95% confidence intervals were estimated using 2,000 bootstrap iterations over the dataset.

To validate this indirect approach, we first inferred the crossover-associated mutation rates. We estimated these to be 0.028 (95% CI=[0.022, 0.034]) and 0.011 (95% CI=[0.005,0.013]) DNMs per paternal and maternal meiosis in these hotspots. In the central 1 kb of hotspots, they represent an elevation of 33-fold (95% CI=[21, 53]) and 46-fold (95% CI=[23, 117]) above local average in fathers and mothers respectively, which are consistent with directly inferred CO-associated mutation rates (*7*), which validates our approach.

We repeated this calculation with another set of hotspots identified using maternal crossovers in the Icelandic population (*7*). The number of DNMs inferred to be due to the recombination machinery is 0.07 (95% CI=[0.03,0.10]), which is consistent with the number inferred from the AA hotspot set (see main text).

We calculated the average number of autosomal crossovers per meiosis was calculated using data from (*7*), which was 52.4 for males and 83.6 for females. We used these values to estimate the total number of DSBs under a model that the DNM rate per DSB is the same regardless of resolution as a crossover or without a crossover, on average (see main text). We estimated that, on average, 352.8 DSBs occur in our hotspot set in male meiosis (it captures ~81% of COs in Icelandic males), implying a DNM rate of 6.6×10^{-4} per DSB. For females, we similarly calculated a DNM rate of 2.0×10^{-4} per DSB (using the alternative hotspot set above, we infer 1.4×10^{-4} per DSB). These data indicate that the *per DSB* mutation rate is 3-5 times higher in fathers than in mothers. In the genome as a whole, 79% of phased DNMs in this cohort were paternal in origin, which is 3.8-fold more frequent than maternally inherited DNMs.

Only a small proportion of potential hotspots in humans undergo a DSB in any given meiosis. The increase in mutation rate observed when averaged across hotspots is due to a higher mutation rate *per DSB* as the vast majority of hotspots do not undergo a break. However, the precise number of sites amongst these hotspots that are used in the population, on average, is unknown for several reasons: (i) the complex relationship between the timing of DNA replication relative to DSBs (*93*) (ii) segregation of hotspot-disrupting alleles at hotspot sites in the population (*94*) (iii) variation in PRDM9 alleles in the population. This implies that the true number of autosomal hotspot sites is of the order of ~20,000 to ~100,000 (the latter being the expected number if all sites are fully replicated with no hotspot-disrupting alleles and only PRDM9 A-allele in the population). To infer the expected proportion of hotspot sites that undergo a break in any given meiosis, we compared the DNM rate per crossover (i.e., sites where a DSB has definitely occurred) with the DNM rate due to all outcomes of recombination (i.e., where the proportion of sites that have undergone a DSB is unknown). Under a parsimonious model in which the probability of a DNM per DSB is the same for crossovers and other outcomes, this provides an estimate of the proportion of hotspot sites that experience a DSB.

From the Icelandic trios, we calculated this value to be 0.89% using the default hotspot size of 2 kb. This implies that ~60,000 sites are available for DSBs on average per meiosis, which is consistent with the range above. This inference is robust to the parameter choices for hotspot size, with the following values inferred for each choice:

- 500 bp: 0.89%
- 1 kb : 0.83%
- 2 kb: 0.89%
- 3 kb: 0.96%

<u>Modelling insertions in unique DNA in recombination hotspots</u>

The sequence of an insertion is assumed to be copied by the polymerase from a template. The template is either the neighbouring sequence (*i.e.* an exact match) or from DNA elsewhere. Mismatches with the neighbouring sequence are assumed to be either due to differences in the template used or copying errors due to the polymerase. To model this process, we propose a mixture model where the probability that the template sequence is the exact neighbouring sequence is given by the mixing parameter $1-\lambda$ (with $0<\lambda< 1$) and the probability of an inexact template is λ. The probability of a copying error due to the polymerase is p and is assumed to be the same for all bases (and independent of neighbouring copying errors). Therefore, the number of errors X in a sequence of length n is distributed binomials with probability mass

$$f_{\text{Exact}}(x|n,p) = \binom{n}{x} p^x (1-p)^{n-x}$$

If the template sequence is not the neighbouring sequence, then we assume that the polymerase uses a similar sequence as the template. To model the mismatches between the template and the exact neighbouring sequence, we assume that the probability of a mismatch at any site of the template sequence is z, where z is beta distributed with parameters α and β

$$f_{\text{Beta}}(z|\alpha,\beta) = \frac{z^{\alpha-1}(1-z)^{\beta-1}}{B(\alpha,\beta)}$$

and $B(\alpha,\beta)$ is the beta function. Including the probability that the polymerase fails to correctly copy the base, we get the probability of a mismatch at each base being

$$\bar{p}(z,p) = p + z - pz$$

with the total number of mismatches being distributed binomial, so that

$$f_{\text{Inexact}}(x|n,\bar{p}) = \binom{n}{x} \overline{p^x} (1-\bar{p})^{n-x}$$

Integrating over z yields the univariate distribution of x

$$f_{\text{Inexact}}(x|n,p,\alpha,\beta) = \int_0^1 f_{\text{Inexact}}\left(x|n,\bar{p}(z,p)\right) f_{\text{Beta}}(z|\alpha,\beta)dz$$

$$= \binom{n}{x}\frac{1}{B(\alpha,\beta)}\int_0^1 (p + z - pz)^x (1 - p - z + pz)^{n-x}\, z^{\alpha-1}(1-z)^{\beta-1} dz$$
$$= \binom{n}{x}\frac{(1-p)^n}{B(\alpha,\beta)}\int_0^1 \left(z + \frac{p}{1-p}\right)^x z^{\alpha-1}(1-z)^{n-x+\beta-1}\, dz$$
$$= \binom{n}{x}\frac{(1-p)^n}{B(\alpha,\beta)}\sum_{j=0}^{x}\binom{x}{j}\left(\frac{p}{1-p}\right)^j B(x-j+\alpha, n-x+\beta)$$

note if *p=0* we obtain the regular beta-binomial distribution. Finally, using the mixture of exact and inexact template sequences, we obtain the probability of *x* mismatches on an insert of length *n* as

$$f_{Mismatch}(x|n,p,\lambda,\alpha,\beta) = (1-\lambda)f_{Exact}(x|n,p) + \lambda f_{Inexact}(x|n,p,\alpha,\beta)$$

This model we call the *Model with Template Switching*. The 4 model parameters were estimated by fitting to 2650 insertions (of length 3 or greater) using Monte Carlo Markov Chain to sample from their posterior distribution. The model was coded in Stan (*95*) and range priors were used for all the parameters. Three MCMC chains were run for 2000 samples, each with a burn-in of 1000 samples and Stan reported that all chains were well mixed (Rhat=1). The posterior distributions for all the parameters were not close to the boundaries of the priors.

As a comparison, we considered two simpler models. The first model, which we call *Polymerase Error Only Model*, assumes that the only source of mismatches is the polymerase copying errors (in the terms of the *Model with Template Switching* this is setting $\lambda = 0$). For consistency of method, we used the same inference procedure (and code) as the *Model with Template Switching* but the used the prior that $\lambda = 0$. The second model, which we call the *Duplication or Random Mode*l, assumes the if the template is not the neighbouring sequence, it is a random sequence (in the terms of the *Model with Template Switching* this is setting $z = 3/4$). Again, for consistency of method, we used the same inference procedure (and code) as the *Model with Template Switching* but used the prior on the coefficients of the beta distribution to be $\alpha=3000$ and $\beta=1000$ (*i.e.* a very narrow distribution centred on $z = 3/4$).

To evaluate the goodness of fit of the 3 models, we look at 3 key statistics which summarise the distribution of mismatches for different lengths of insertions (Fig. S10 A-C). The first two statistics are the mean and the variance of the number of mismatches. The mean number of mismatches grows linearly with the length of the insertion and all models successfully capture this feature. The variance of the number of mismatches is linear with the length of the insertions for small insertions, however, increases more rapidly for longer insertions (approximately quadratically). The *Polymerase Error Only Model* is not able capture this feature of the variance in mismatches. The *Duplication or Random Model* captures the basic feature, however, overestimates the variance in mismatches for larger insertions. The *Model with Template Switching* successfully captures the variance in mismatches for all lengths of insertions. The third statistic is the proportion of insertions with no mismatches. This declines from 80% for the shortest insertions to about 60% for insertions of length 10 and then very gradually declines for longer insertions. Both the *Polymerase Error Only Model* and the *Duplication or Random Model* fail to capture this effect, which is due to the models requiring a very high polymerase error rates (>5%) to fit the data (*i.e.* fit the mean number of

mismatches). The *Model with Template Switching* successfully captures this feature of the data.

In summary, the *Model with Template Switching* successfully captures the detail of the distributions of mismatches for insertions of different lengths, despite containing only 4 parameters. Under this model, we can now examine what the distribution of mismatches in the insertions implies about the mismatches in the template and the polymerase error rate (Fig. S10 E-F). The model implies that the template is an exact match 63% (95% CI=[60%, 66%]) of the time, and the probability of template matching at more than half of the bases is 87% (95% CI=[86%, 89%]), which is consistent with template-switching. The model implies a polymerase error rate of 1.2% (95% CI=[1.0%,1.5%]).

Supplementary Text

Factors influencing de novo single-base substitution and indel mutation rates in unique DNA and TR sequences

For each mutation type, we modelled the number of autosomal mutations with three distinct generalized linear models using a quasipoisson link function with the following predictor variables:

Model 1. Hotspot intensity (and TR copy number in case of TR indels)

Model 2. Hotspot intensity and background mutation rate (and TR copy number in case of TR indels)

Model 3. Hotspot intensity, background mutation rate, and whether the hotspot is close to the telomere (defined as being within 1 Mb of the telomeres as reported in Hg38) and TR copy number (in case of TR indels)

The results are summarized in Table S1 and we highlight the following inferences:

- Mutation rates in recombination hotspots are correlated with hotspot intensity, over and above background mutation rate, for single-base substitutions, insertions and deletions in both unique and TR DNA.
- There is significant variation in the background mutation rate for different types of mutations in different sequence contexts. In TR sequences, for example, the TR copy number explains nearly the entire background mutation rate for indels.
- The mutation rate in telomere-proximal hotspots is higher, over and above what is expected from the background mutation rate in these regions and the rate expected from hotspots with similar DMC1 intensity elsewhere in the genome. Increase in minisatellite instability, particularly at telomere proximal recombination hotspots, is known (*51*) and our analyses quantify and extend that understanding to minisatellite and microsatellites genome-wide. Furthermore, these analyses indicate that recombination hotspots near telomeres have higher mutation rates more generally, including in unique DNA and for both indels and single-base substitutions.

Leveraging Alu and ERVL-MaLR sequences to characterize the impact of meiotic break repair on mutagenesis

To check that elevated mutation rates are due to the recombination machinery per se (as opposed to, say, sequence composition), we examined Alu and THE1 retrotransposon families. They have tens to hundreds of thousands of copies throughout the genome. Whereas most of these elements do not harbour hotspots, a subset of them have intact PRDM9-binding motifs due to small differences from the canonical retrotransposon sequence. Therefore, we compared elements that overlap hotspots relative to those that do not (*41*). These data show higher indel rates with a bias towards insertions in hotspot-overlapping elements (Fig. S6A-D), demonstrating that the recombination machinery itself is responsible for elevated mutagenesis.

Furthermore, we found that repetitive sequences overlapping hotspots are longer on average than those that do not overlap hotspots. For example, Alu elements that overlap hotspots are 283.0 bp long on average, compared with 260.2 bp on average for Alu elements that do not. Therefore, we checked whether our observations of higher mutagenesis are driven by a

genuine increase in mutation rate per base as opposed to the longer length of the elements. In order to do so, we compared polymorphisms in subsets of Alu and ERVL-MaLR elements matched for element size. We observed the following:

For Alu elements:

- We restricted to elements of size 265-325 bp. The mean size of elements overlapping hotspots was 299.0 bp and 298.6 bp for elements not overlapping hotspots.
  - The mean number of insertions is 9.146 in elements overlapping hotspots and 4.929 in elements not overlapping hotspots.
  - The mean number of deletions is 7.292 in elements overlapping hotspots and 4.468 not overlapping hotspots.

We conclude that increase in indels in hotspot-overlapping Alu elements is also observed in size-matched elements is not an artefact of their longer length.

For ERVL-MaLR elements (which include THE1B elements):

- We restricted to elements of size 340 to 375 bp. The mean size of elements overlapping hotspots was 358 bp and 359 bp for elements not overlapping hotspots.
  - The mean number of insertions is 2.788 in elements overlapping hotspots and 1.338 in elements not overlapping hotspots
  - The mean number of deletions is 3.547 in elements overlapping hotspots and 2.866 in elements not overlapping hotspots
- THE1B. We again restricted to elements of size 340 to 375 bp.
  - The mean number of insertions is 2.557 in elements overlapping hotspots and 1.177 in elements not overlapping hotspots
  - The mean number of deletions is 3.538 in elements overlapping hotspots and 2.781 in elements not overlapping hotspots
- THE1A. We again restricted to elements of size 340 to 375 bp.
  - The mean number of insertions is 3.814 in elements overlapping hotspots and 1.196 in elements not overlapping hotspots)
  - The mean number of deletions is 3.814 in elements overlapping hotspots and 2.890 in elements not overlapping hotspots

We conclude that increase in indels in hotspot-overlapping in ERVL-MaLR/THE1A/THE1B elements is also observed in size-matched elements and not an artefact of their longer length.

These data indicate that the longer length of hotspot-overlapping elements can be explained by the bias towards insertions relative to deletions.

<u>Rate and pathogenicity of single-base substitutions and indels in recombination hotspots</u>

To assess the pathogenicity of mutations within hotspots relative to those mutations elsewhere in the genome, we counted the number of extremely rare SNPs and indels in gnomAD (that also passed all the filters as detailed above) whose VEP (Variant Effect Prediction) was assessed to be ‘HIGH’ (leading to protein truncation, loss of function or

triggering nonsense mediated decay). We refer to them collectively as LOF (loss of function) for simplicity.

- LOF SNPs:
    - Inside hotspots (autosomes and X chromosome): 5729
    - Genome as a whole (autosomes and X chromosome): 185905

- All SNPs:
    - Inside hotspots (autosomes and X chromosome): 7671768
    - Genome as a whole (autosomes and X chromosome): 341221825

- LOF Indels:
    - Inside hotspots (autosomes and X chromosome): 5905
    - Genome as a whole (autosomes and X chromosome): 182726

- All Indels:
    - Inside hotspots (autosomes and X chromosome): 1424928
    - Genome as a whole (autosomes and X chromosome): 55980422

The total sequence length in hotspots used for these assessments is 56,130,000 (28,065 autosomal or X chromosome hotspots), with the sequence length for the human genome in the autosomes and the X chromosome 3,042,506,734 bp.

Note that the ratio of pathogenicity of SNPs as inferred from these data (i.e., 38% (95% CI=[35%, 42%]) higher) is subtly but significantly higher than the corresponding ratio for indels (28% (95% CI=[25%, 31%]). This suggests that the relationship between variant size and pathogenicity is subtly distinct for hotspots than elsewhere in the genome. This is consistent with expectations from the over-representation of exons in hotspots: for a variant of any given size, the chance of impacting an exon is higher for variants in hotspots.

<u>ClinVar analysis of hotspot-overlapping exons</u>

To calculate the excess in pathogenic mutations due to the recombination machinery, we restricted to genes with multiple exons, such that at least one exon overlapped a hotspot and at least one exon did not overlap any hotspots (n=3,434). We counted the number of SNPs, indel breakpoints, and SV breakpoints originating in each exon. For exons that overlap hotspots (+/- 1kb from hotspot centres), we included the segment of the exon that overlaps with the hotspot. Amongst these genes, the majority (n=2,136) did not have any exonic pathogenic mutations in the ClinVar catalogue. (This is not surprising as fewer than a third of human genes have a pathogenic mutation in the catalogue.)

For the remaining 1,298 genes, we counted the total number of pathogenic mutations, as detailed above, for hotspot-overlapping and non-hotspot-overlapping exons, respectively. We also added up the sizes of these regions. We thereby calculated the number of mutations per base in each of these categories. The p-values and 95% CIs were estimated using bootstrap (100,000 iterations). The 41% increase we observed is consistent with, and intermediate to, the enrichment of the different types of variants in hotspots at the 2 kb scale (SNPs, indels and SVs).

For each gene that has at least one pathogenic variant in a hotspot-overlapping exon (n=514), we assessed the evidence for excess pathogenic mutations. We performed Fisher's exact test with the following contingency table: Pathogenic mutations in hotspot-overlapping exons, Pathogenic mutations in non-overlapping exons; Size of hotspot-overlapping exons, Size of non-overlapping exons. The genes reported in Table S3 have p-value < 9.8×10^{-5} (p-value threshold of 0.05 after Bonferroni correction for 514 tests).

Note that structural variants reported in ClinVar have been identified using a range of technologies, not all of which rely on DNA sequencing. Imprecision in calling SV breakpoints would be predicted to add noise, thereby reducing power to attribute breakpoints accurately to hotspots and/or exons. To investigate this, we restricted the definition of a hotspot to ±100 bp from hotspot centres and ±250 bp from hotspot centres.

For exonic regions ±100 bp from hotspot centres, we observed that the excess of pathogenic mutations is 92% (95% CI=[49%, 249%]) and for exonic regions ±200 bp from hotspot centres it is 58% (95% CI=[27%, 98%]), respectively. This is consistent with expectations as the mutation rate is higher closer to hotspot centres.

These analyses suggest that loss of power from imprecision, if any, in SV calls in ClinVar is not sufficient to ablate the signal of elevated pathogenic mutations due to meiotic breaks. We conclude that our overall finding of excess pathogenic mutations in hotspot-overlapping exonic regions is robust to use of these data.

Provenance of SVs in recombination hotspots

SV size distribution

We examined the distribution of SV sizes in both gnomAD-SV and deCODE-SV datasets. Both SV deletions and insertions smaller than 2 kb are over-represented in hotspots (Fig. S12D-G). For gnomAD-SV the odds ratio for increase in SV deletions smaller than 2 kb is 1.9 ($p=1.3 \times 10^{-20}$) and for insertions it is 2.9 ($p=6.8 \times 10^{-34}$). The results from deCODE-SV are consistent with gnomAD: for deletions it is 1.8 ($p=7 \times 10^{-4}$) and insertions it is 1.6 (p=0.13) (Fisher's exact test was used for all of these tests). The combined p-value is reported in the main text.

Number of SVs in hotspots is correlated with hotspot intensity and background mutation rate

The number of SVs is correlated with hotspot intensity and background rate for both the X chromosome and the autosomes. We modelled them with multiple regression separately for X and autosomal hotspots. For the X chromosome, the p-value of association with hotspot intensity is $p=2 \times 10^{-83}$ and with background mutation rate is 3×10^{-56}. The respective values for the autosomes are $p=9 \times 10^{-13}$ and p=0, respectively.

SVs with breakpoints in Alu elements

Amongst autosomal SV deletions larger than 2 kb with a breakpoint within 100 bp of a hotspot centre, we observed an unexpectedly large proportion with breakpoints in Alu elements (Fig. 8A) (35% vs 9% expected; 95% CI=[20%, 54%], $p=2 \times 10^{-5}$, n=34). To investigate the properties of SV deletions in Alu elements, we considered all SV deletions

that have their hotspot-proximal breakpoint in an Alu element but are too large to be within a single element (>=400 bp) (n=60). The hotspot-distal breakpoint overlapped another Alu element in the vast majority of cases (90%, 54/60). The orientation of both elements was the same in every case (54/54, $p=1.1x10^{-16}$). The vast majority (92%) exhibited microhomology at breakpoints (median=16.5 bp) (Fig. S12F). SVs with similar properties have previously been reported around the *SPAST* gene (*96*).

Multiple mechanisms can explain deletions between regions of high sequence similarity. Apart from TMEJ, two possibilities are NAHR and single-strand annealing (SSA), a pathway that requires longer stretches of homology than are typical for TMEJ (>50 bp) (*27*, *72*). The median homology between Alu elements underlying the SV deletions analysed above is 0.83 and 0.76 over ±50 bp and ±100 bp from the breakpoints, respectively (Fig. S12G). Whilst the observed microhomology is consistent with TMEJ, the overall homology is lower than is typical for NAHR or SSA for most of these SVs.

The ssDNA required for TMEJ could be generated by break-induced replication (BIR) in SVs longer than the extent of resection (we have seen evidence for BIR also in point mutations, Fig. 6C-D). However, an alternative pathway called microhomology-mediated break induced replication (MMBIR) (*27*) is also possible. It has been proposed that TMEJ and MMBIR may be related pathways, with MMBIR being mediated in part by Polθ (*72*)).

There is insufficient data to characterize SVs larger than 2 kb in non-repetitive (unique) DNA.

Polarisation test for SV deletions between hotspot centres.

As reported in the main text, we observed 11 SV deletions larger than 2kb in length that had both breakpoints near distinct hotspot centres (within +/- 200 bp of centres). They constituted 8% of deCODE and 1% of gnomAD SV deletions of this size scale that had at least one breakpoint within 200 bp of a hotspot centre. The difference between deCODE and gnomAD is consistent with lower breakpoint resolution of gnomAD. This is extremely unlikely to occur by chance if hotspot density were unvarying in the genome. However, we observed that SVs with breakpoints in hotspots were predominantly near telomeres and on the X chromosome. Therefore, we sought to check that this phenomenon was not simply chance overlap due to higher hotspot density in these regions. We performed a “polarization test” to address this possibility.

Specifically, for each SV deletion > 2 kb, of length, say, l, and breakpoints (s, e), we generated two matched hypothetical SV deletions with breakpoints ($s-l, s$) and ($e, e+l$), respectively. We then counted the number of SV deletions that had both breakpoints within 200 bp of hotspot centres in the actual data and the matched dataset. The p-value reported in the main text is from Fisher’s exact test comparing these counts. Interestingly, the PRDM9 motif orientation was the same in both flanking hotspots in 10 out of the 11 SVs (p=0.006).

Why do breaks cluster in hotspots near functionally important regions of the genome?

In many species, including humans and mice, repair of meiotic DNA breaks achieves two distinct, albeit related, functions. Firstly, it enables numerous interactions between homologous chromosomes, enabling them to pair up (synapsis). Secondly, a subset of these interactions result in the formation of crossovers, which are necessary for correct segregation of chromosomes into haploid gametes (*1*).

Many lines of evidence suggest that DNA breaks occur in vast excess of the number of crossovers in order to ensure synapsis. For example, *Spo11*$^{+/-}$ mice have fewer breaks (*86*). Their chromosomes fail to synapse, leading to meiotic arrest and total infertility (*86*). In some organisms, e.g., *C. elegans.*, chromosomes pair using a different mechanism that does not rely on DNA breaks (*97*). *C. elegans.* chromosomes have only 1-2 DNA breaks, one of which is resolved with the obligatory crossover (*97*).

Whilst these studies provide a rationale for large numbers of DNA breaks, they don't explain the concentration of breaks in hotspots. A body of work on the protein PRDM9 shows that successful synapsis requires binding of PRDM9 not only to the DSB site, but also to the corresponding site on the homologous chromosome that is used as a template to repair the break (*9*, *38*, *83*). A recent preprint modelling the evolution of PRDM9 suggests that new PRDM9 alleles provide an advantage by limiting the number of binding sites, thus reducing the search space and accelerating synapsis (*98*).

This in turn leads to the question about why these hotspots are enriched near exons. Another set of human PRDM9 alleles (C-like alleles) bind distinct sites, but these are also enriched near exons to an extent similar to the A-like alleles (Fig. 5A, 7A). Mouse hotspots are also enriched in exons (*38*). In contrast, in many species (e.g., yeast and birds), recombination is not positioned by PRDM9. Many breaks in these species take place near transcription start sites (TSSs). As with exons, mutations near TSSs are likely to prove deleterious.

It is possible that the chromatin in these regions is conducive to break repair because of increased accessibility or other factors (*87*), which may increase the likelihood of successful synapsis and meiosis. If this is the case, it would imply that the increase in fertility outweighs the burden of genetic disease from mis-repair of DSBs.

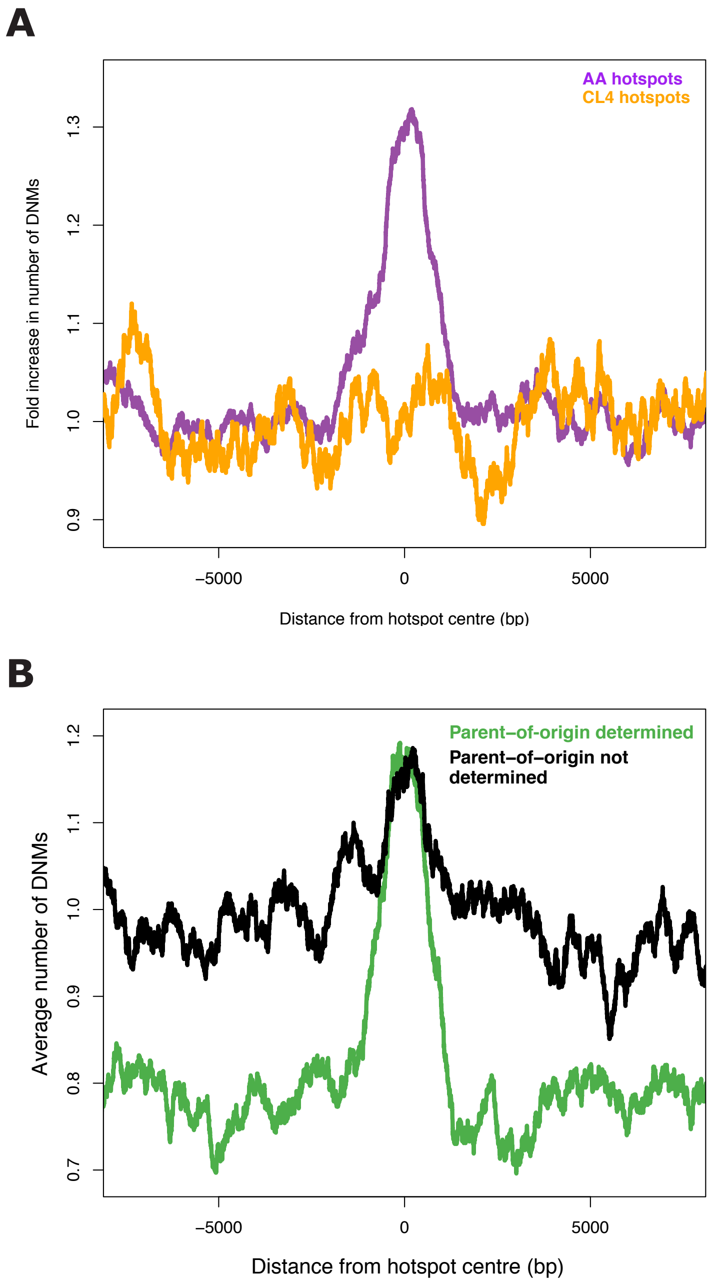

Fig. S1. De novo mutations in human recombination hotspots. (A) To check that the DNM rate increase in hotspots is a consequence of recombination-associated processes (as opposed to, say, a priori properties of sites that are bound by PRDM9), we compared the average number of DNMs in hotspots activated by the PRDM9 A-allele (purple) relative to hotspots activated by the PRDM9 C and L4 alleles (orange). PRDM9 alleles with binding properties similar to the A-allele comprise the vast majority (~95%) of the PRDM9-variation in northern Europeans. In contrast, C-type alleles (which include the L4 allele) are rare in European populations and bind a distinct set of hotspots. We observed no increase in DNM rate in the Icelandic cohort in CL4 hotspots (orange), in contrast with AA hotspots (purple). This demonstrates that DNM rate in the vicinity of a hotspot is a good estimate of the DNM rate inside the hotspot in the absence of recombination. **(B)** Parent-of-origin was determined in a greater proportion of DNMs inside hotspots than those outside, indicating higher power to phase mutations in hotspots (despite breakdown of linkage disequilibrium in hotspots). It is likely that this is due to the excess of rare and extremely rare variants in hotspots (see Figure 2). We corrected for this effect in sex-specific DNM rate measurements to prevent over-counting of DNMs in hotspots.

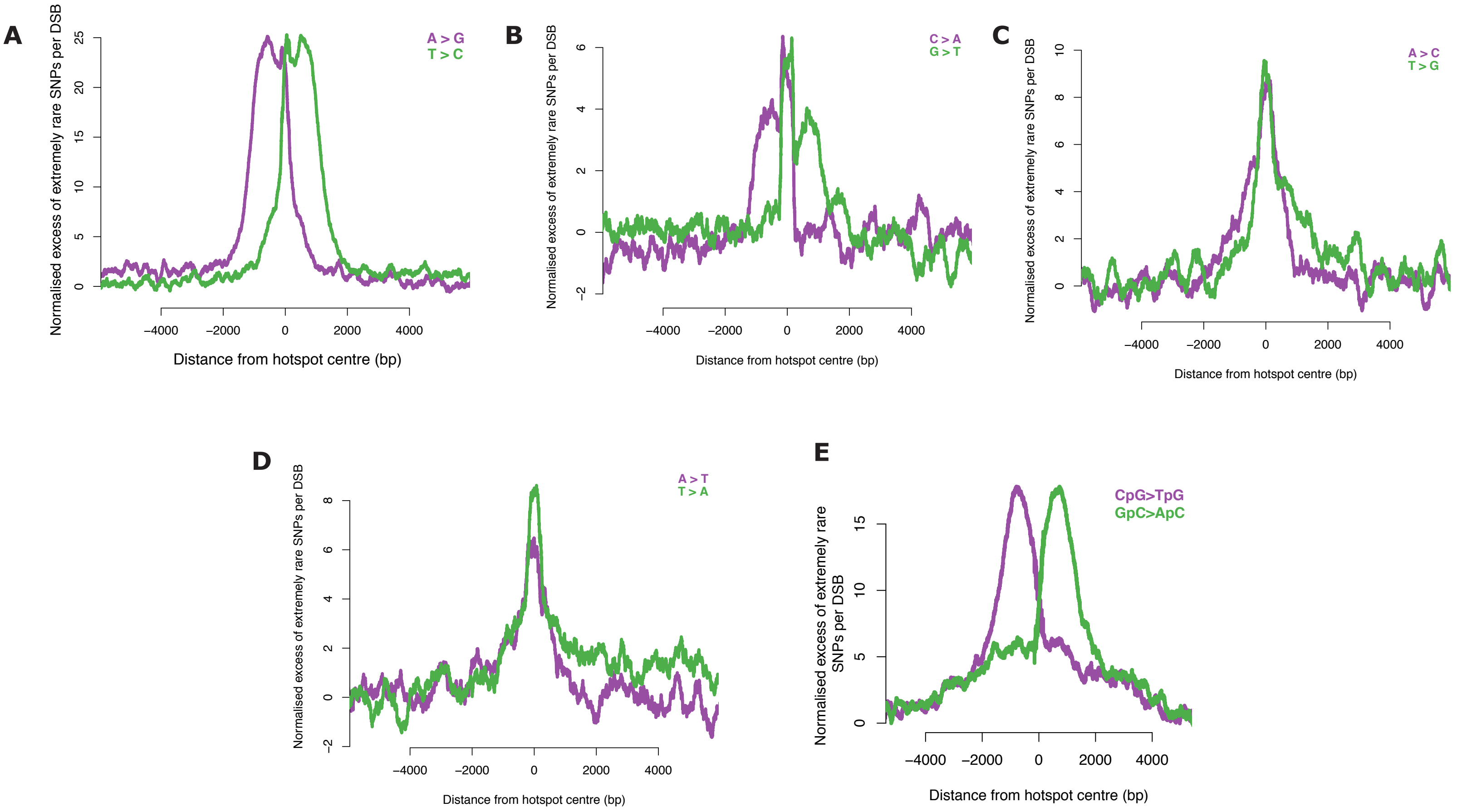

Fig. S2. Point mutations in human recombination hotspots. (A) Fold excess in the number of A>G and T>C per 'A' and 'T' base, respectively (allele frequency<10^{-3}, 200 bp moving window). The figure corrects for and is robust to differences in sequence composition in and around hotspots. **(B)** As (A) but for C>A and G>T mutations (400 bp moving window) **(C)** As (B) but for A>C and T>G mutations. **(D)** As (B) but for A>T and T>A mutations. **(E)** As (A) but for CpG>TpG and GpC>ApC mutations.

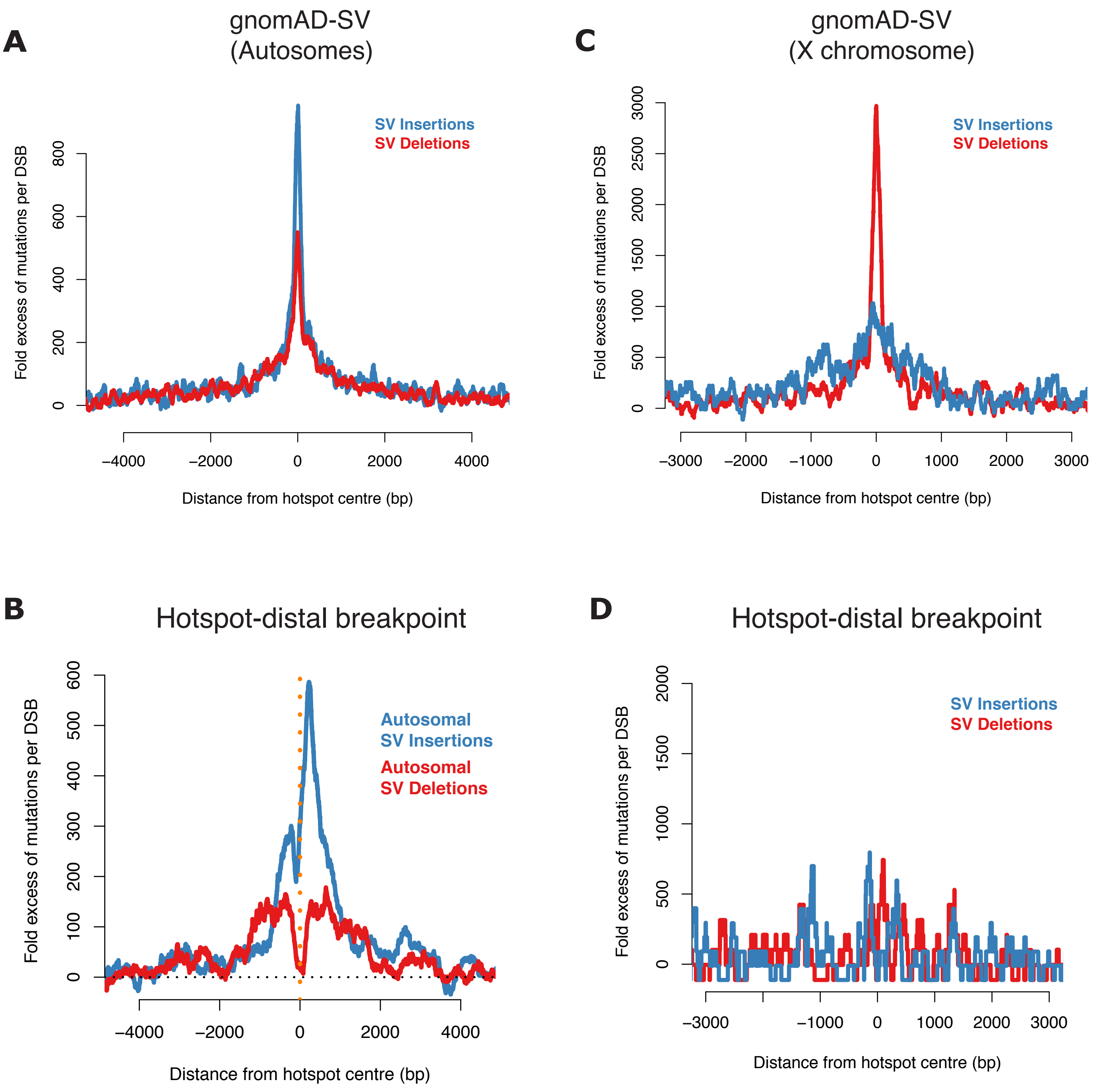

Fig. S3. SV breakpoints relative to human recombination hotspots. **(A)** Fold excess in the number of hotspot-proximal SV breakpoints (allele frequency < 10^{-2}) per DSB in gnomAD-SV relative to autosomal hotspots (100 bp moving window). **(B)** Fold excess in the number of hotspot-distal SV breakpoints (allele frequency < 10^{-2}) per DSB in deCODE-SV relative to autosomal hotspots (300 bp moving window). **(C)** As (A) but for the X chromosome. **(D)** As (B) but for the X chromosome.

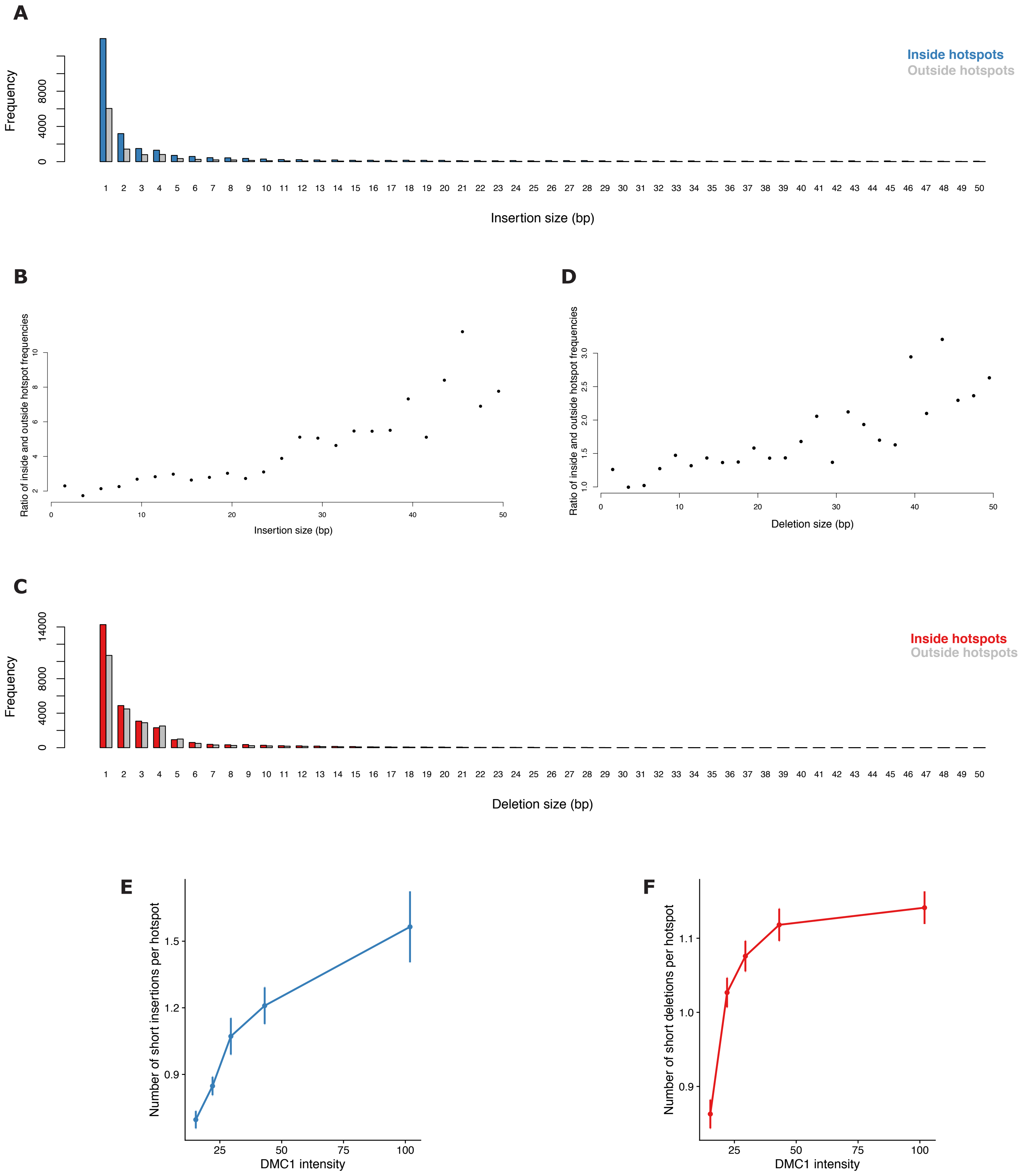
A
Frequency
0
4000
8000
Insertion size (bp)
1 2 3 4 5 6 7 8 9 10 11 12 13 14 15 16 17 18 19 20 21 22 23 24 25 26 27 28 29 30 31 32 33 34 35 36 37 38 39 40 41 42 43 44 45 46 47 48 49 50
Inside hotspots
Outside hotspots
B
Ratio of inside and outside hotspot frequencies
2 4 6 8 10
0 10 20 30 40 50
Insertion size (bp)
D
Ratio of inside and outside hotspot frequencies
1.0 1.5 2.0 2.5 3.0
0 10 20 30 40 50
Deletion size (bp)
C
Frequency
0
4000
8000
14000
Deletion size (bp)
1 2 3 4 5 6 7 8 9 10 11 12 13 14 15 16 17 18 19 20 21 22 23 24 25 26 27 28 29 30 31 32 33 34 35 36 37 38 39 40 41 42 43 44 45 46 47 48 49 50
Inside hotspots
Outside hotspots
E
Number of short insertions per hotspot
0.9
1.2
1.5
25 50 75 100
DMC1 intensity
F
Number of short deletions per hotspot
0.9
1.0
1.1
25 50 75 100
DMC1 intensity

Fig. S4.

Properties of indels in recombination hotspots with both breakpoints in unique DNA. (A) Histogram of number of insertions that have a breakpoint within 100 bp of hotspot centres (blue) or 8-10 kb from it (grey, rescaled to the average number per 200 bp to facilitate comparison). **(B)** Ratio of the number of insertions per base within 100 bp of hotspot centres relative to 8-10 kb from it for different insertion sizes (rescaled as for (A)) **(C)** As (A) but for deletions **(D)** As (B) but for deletions. **(E)** Number of insertions in unique DNA relative to DMC1 intensity. Hotspots were binned into 5 equal bins ordered by DMC1 intensity and the average number of insertions within 100 bp of hotspot centres is shown. Error bars show one standard error. **(F)** As (E) but for deletions.

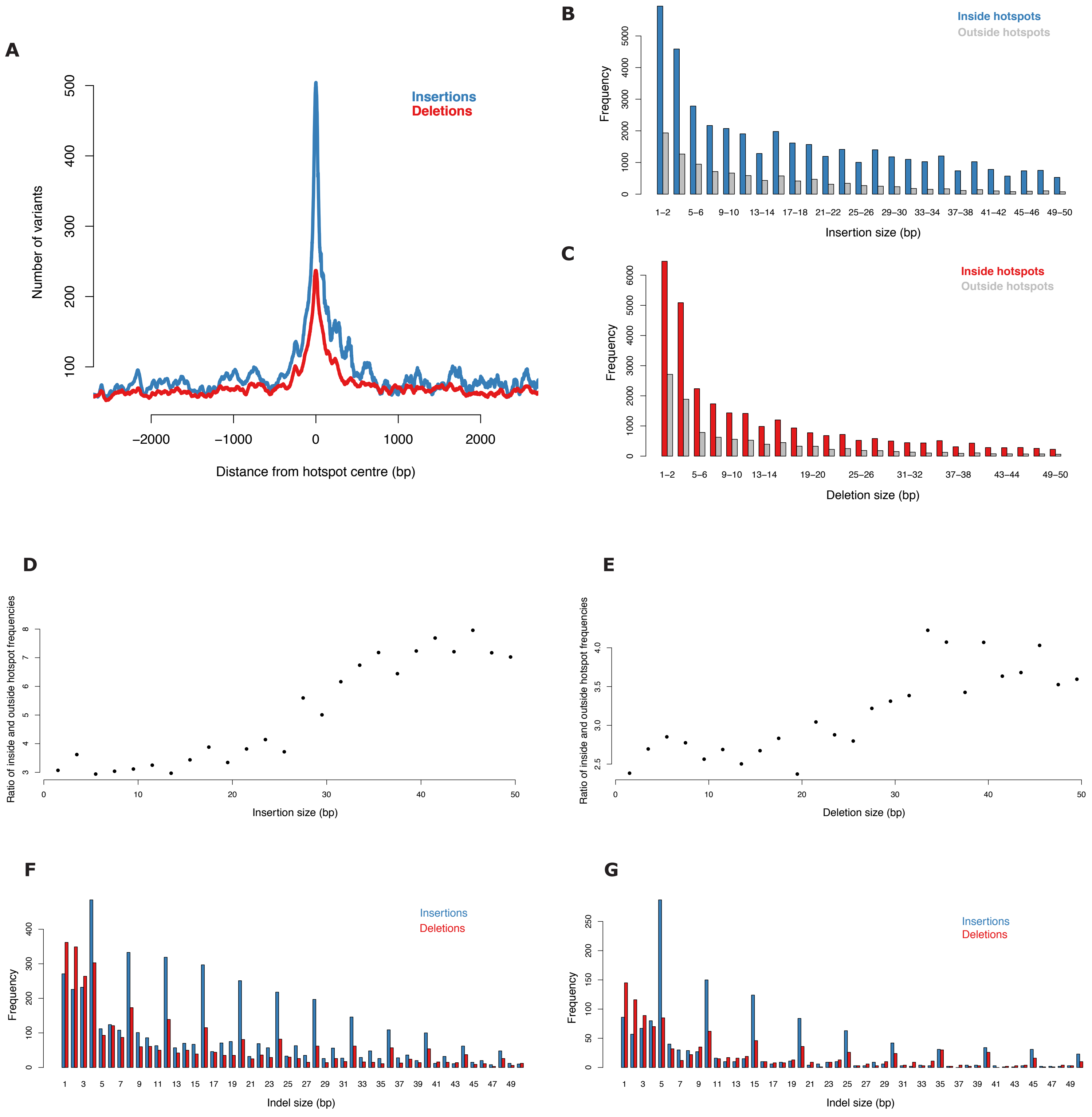
A
Insertions
Deletions
Number of variants
100
200
300
400
500
−2000
−1000
0
1000
2000
Distance from hotspot centre (bp)
B
Inside hotspots
Outside hotspots
Frequency
0
1000
2000
3000
4000
5000
1–2
5–6
9–10
13–14
17–18
21–22
25–26
29–30
33–34
37–38
41–42
45–46
49–50
Insertion size (bp)
C
Inside hotspots
Outside hotspots
Frequency
0
1000
2000
3000
4000
5000
6000
1–2
5–6
9–10
13–14
19–20
25–26
31–32
37–38
43–44
49–50
Deletion size (bp)
D
Ratio of inside and outside hotspot frequencies
3
4
5
6
7
8
0
10
20
30
40
50
Insertion size (bp)
E
Ratio of inside and outside hotspot frequencies
2.5
3.0
3.5
4.0
0
10
20
30
40
50
Deletion size (bp)
F
Insertions
Deletions
Frequency
0
100
200
300
400
1
3
5
7
9
11
13
15
17
19
21
23
25
27
29
31
33
35
37
39
41
43
45
47
49
Indel size (bp)
G
Insertions
Deletions
Frequency
0
50
100
150
200
250
1
3
5
7
9
11
13
15
17
19
21
23
25
27
29
31
33
35
37
39
41
43
45
47
49
Indel size (bp)

Fig. S5.

Properties of indels in Tandem Repeat (TR) DNA relative to recombination hotspots.
(A) Number of indels in TRs relative to hotspots. Hotspot-proximal breakpoint is shown. **(B)** Histogram of number of insertions in a 200 bp window that have a breakpoint either within 100 bp of hotspot centres (blue) or 8-10 kb from it (grey). **(C)** As (B) but for deletions **(D)** Ratio of the number of insertions per base within 100 bp of hotspot centres relative to 8-10 kb from it for different insertion sizes. **(E)** As (D) but for deletions **(F)** Histogram of insertion (blue) and deletion (red) sizes in TRs with 4 bp periodicity that have a breakpoint within 100 bp of a hotspot centre **(G)** As (F) but for TRs with 5 bp periodicity.

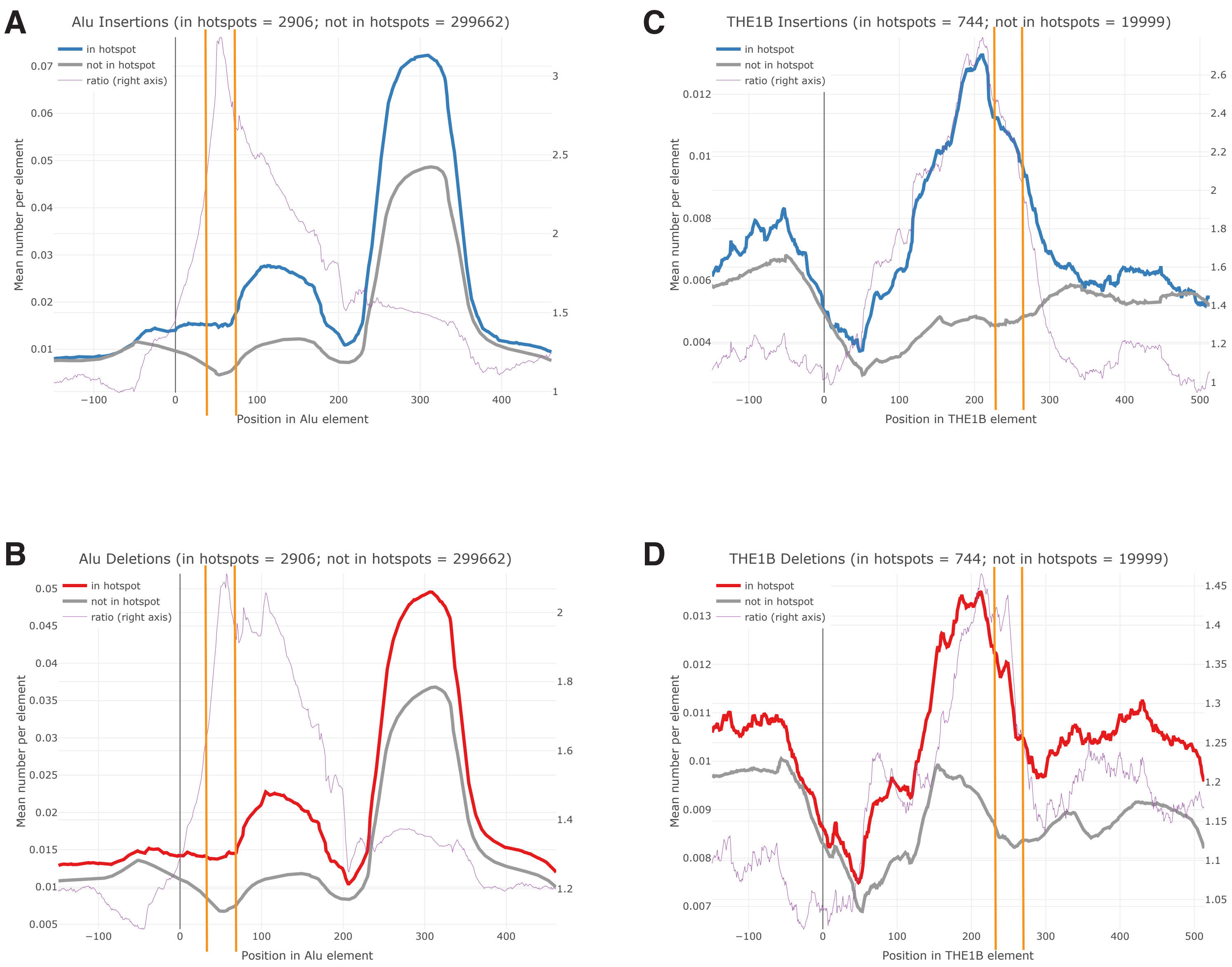

Fig. S6. Comparison of indel frequencies in Alu and THE1B elements with and without overlapping hotspots. (A) Comparison of insertion rates in Alu elements that overlap recombination hotspots (blue) relative to those that do not (grey). The ratio is shown in purple and the inferred PRDM9 binding site is between the vertical orange lines. For computational tractability, we randomly sampled the Alu elements that do not overlap hotspots (1.12 million) down to ~300,000. **(B)** As (A) but for deletions **(C)** As (A) but for insertions in THE1B elements; down-sampling was not required **(D)** As (C) but for deletions.

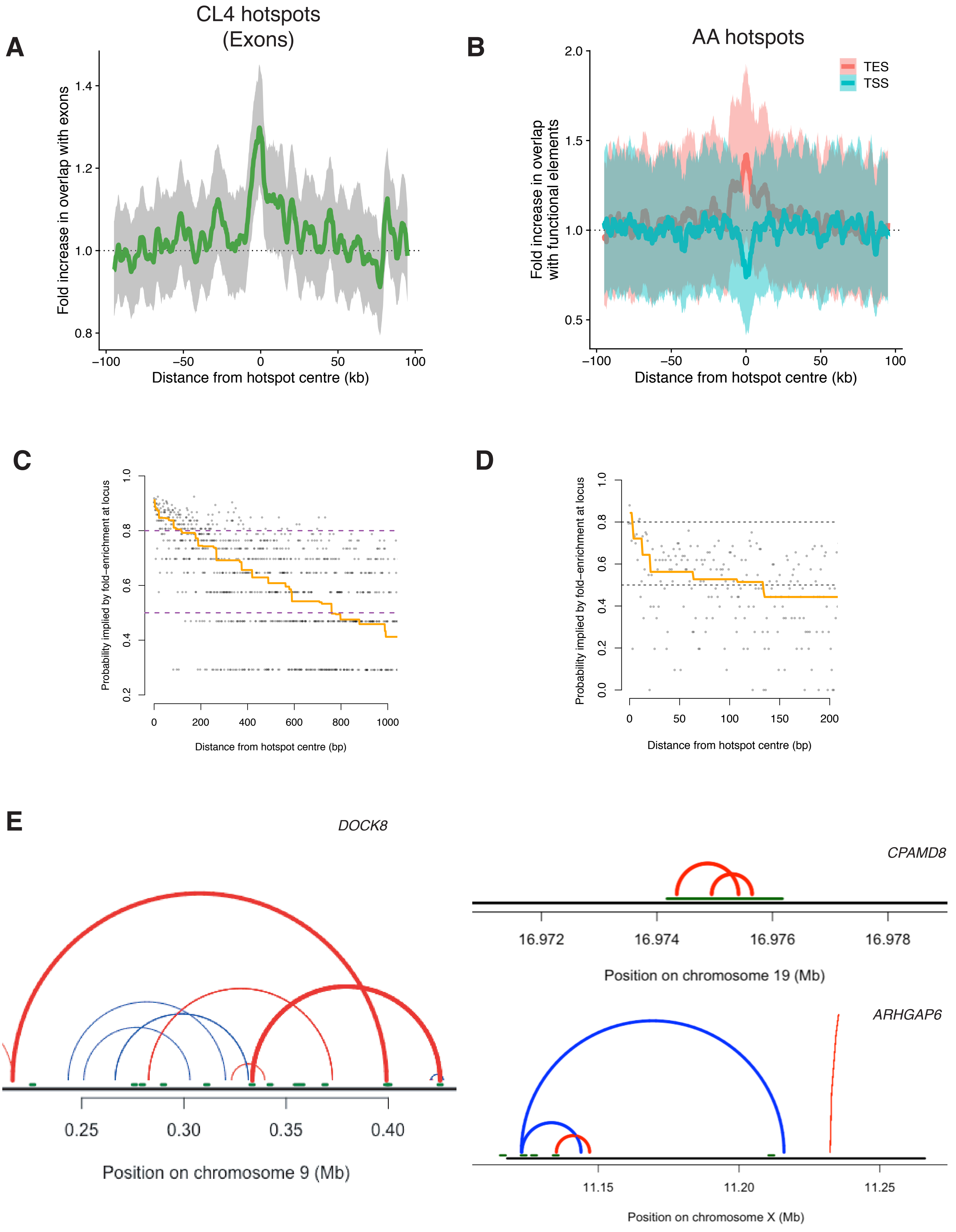
A
CL4 hotspots
(Exons)
Fold increase in overlap with exons
Distance from hotspot centre (kb)
B
AA hotspots
TES
TSS
Fold increase in overlap with functional elements
Distance from hotspot centre (kb)
C
Probability implied by fold−enrichment at locus
Distance from hotspot centre (bp)
D
Probability implied by fold−enrichment at locus
Distance from hotspot centre (bp)
E
DOCK8
Position on chromosome 9 (Mb)
CPAMD8
Position on chromosome 19 (Mb)
ARHGAP6
Position on chromosome X (Mb)

Fig. S7.

Disease impacts of SVs originating in recombination hotspots. (A) Enrichment of exons near human hotspots activated by CL4 PRDM9 alleles (green). The whole exon body was included for each exon. 95% confidence intervals are shown in grey (3 kb moving window). **(B)** As (A) but for transcription start sites (TSS, blue) and transcription end sites (TES, pink) around AA hotspots. 50 bp upsteam and downstream of the elements were included. 95% confidence intervals are shown in the respective colours. PRDM9-bound hotspots are known to be depleted in transcription start sites [Brick et al Nature 2012]. **(C)** Probability that an SV has arisen as a result of meiotic break repair inferred from observed over-representation of SV breakpoints. This is calculated at each bp from the hotspot centre based on the breakpoint enrichment observed in deCODE SVs at that distance (grey). We regressed a piece-wise monotonic function to the data with a node point at every base pair (orange) (Methods). **(D)** As (C) but for indels. **(E)** Further examples of predicted gene loss-of-function SVs. Gene bodies are in black, hotspots are marked in green and insertions (blue) and deletions (red) are indicated by arcs. SVs with a breakpoint in a hotspot are shown with thicker arcs. The genes (and their associated diseases) shown here: DOCK8 (Combined immunodeficiency due to DOCK8 deficiency), CPAMD8 (Anterior Segment Dysgenesis), and ARHGAP6 (Amelogenesis Imperfecta).

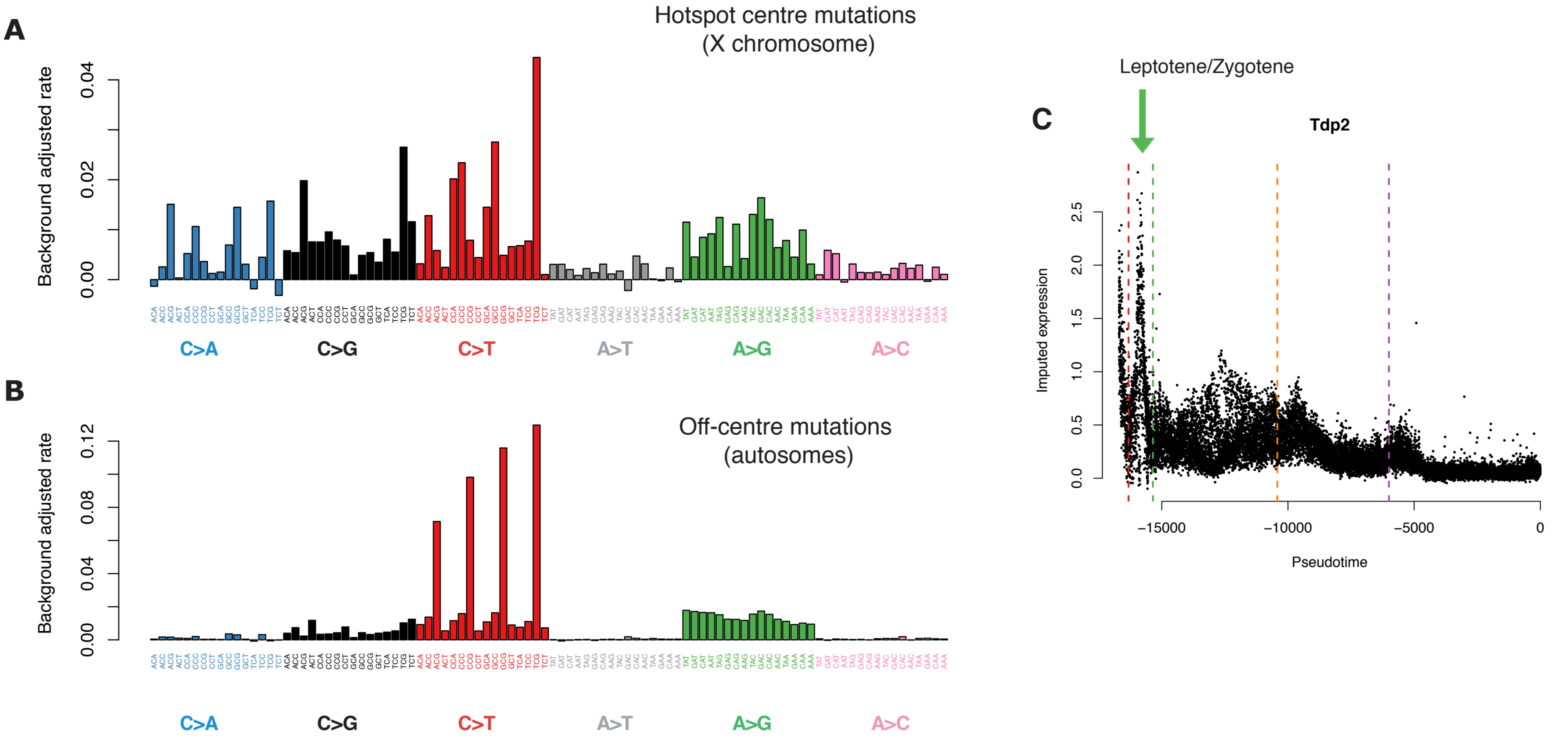

Fig. S8. Mechanisms of point mutations in human recombination hotspots. (A) The number of mutations observed per base in the central region of X chromosome hotspots (+/- 50 bp from the centre of the PRDM9 binding motif) after subtracting the expected rate from local background. **(B)** As (A) but for the the off-centre peaks in autosomal hotspots. **(C)** Time course of the expression of Tdp2 imputed via single-cell RNA-seq data from mouse testis cells. Processing of breaks by TDP2 enables non-homologous end-joining [Johnson et al Nature 2021, Prieleret al Nature 2021]. Dashed lines mark approximate transition points in meiotic stages from left to right: Spermatogonia, Leptotene/Zygotene, Pachytene, Meiotic divisions, Spermiogenesis. Peak expression is observed in Leptotene/Zygotene (green arrow), which is the period in which programmed DSBs are induced.

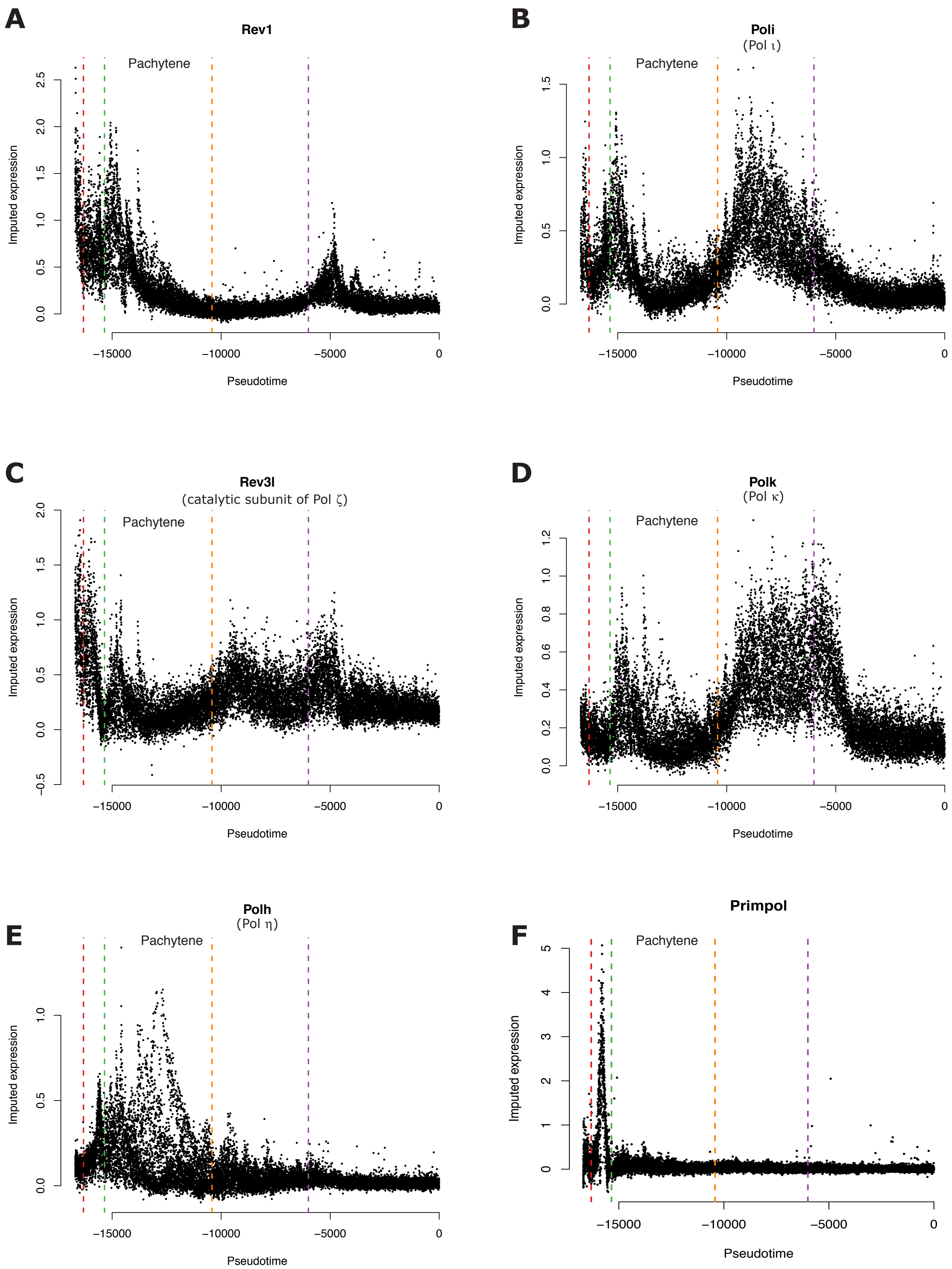
A
Rev1
Pachytene
Imputed expression
0.0
0.5
1.0
1.5
2.0
2.5
−15000
−10000
−5000
0
Pseudotime
B
Poli
(Pol ι)
Pachytene
Imputed expression
0.0
0.5
1.0
1.5
−15000
−10000
−5000
0
Pseudotime
C
Rev3l
(catalytic subunit of Pol ζ)
Pachytene
Imputed expression
−0.5
0.0
0.5
1.0
1.5
2.0
−15000
−10000
−5000
0
Pseudotime
D
Polk
(Pol κ)
Pachytene
Imputed expression
0.0
0.2
0.4
0.6
0.8
1.0
1.2
−15000
−10000
−5000
0
Pseudotime
E
Polh
(Pol η)
Pachytene
Imputed expression
0.0
0.5
1.0
−15000
−10000
−5000
0
Pseudotime
F
Primpol
Pachytene
Imputed expression
0
1
2
3
4
5
−15000
−10000
−5000
0
Pseudotime

Fig. S9.
Time-courses of expression of Translesion Synthesis (TLS) Polymerases (REV1, POLι, POLζ, POLκ, POLη , and PRIMPOL imputed via single-cell RNA-seq data from mouse testis cells. Dashed lines mark approximate transition points in meiotic stages from left to right: Spermatogonia, Leptotene/Zygotene, Pachytene, Meiotic divisions, Spermiogenesis. DNA break induction commences in Leptotene and most breaks are repaired by the end of Pachytene. Whilst some TLS polymerase have high expression levels in Pachytene (Rev1, Polh, Polk, Poli), others (Primpol, Rev3l) have higher expression levels in Leptotene/Zygotene. The precise mutational composition of individual hotspots may be impacted by the timing of break repair, e.g., the excess of C>G mutations attributed to REV1 is consistent with late repair of X chromosome breaks in male meiosis.

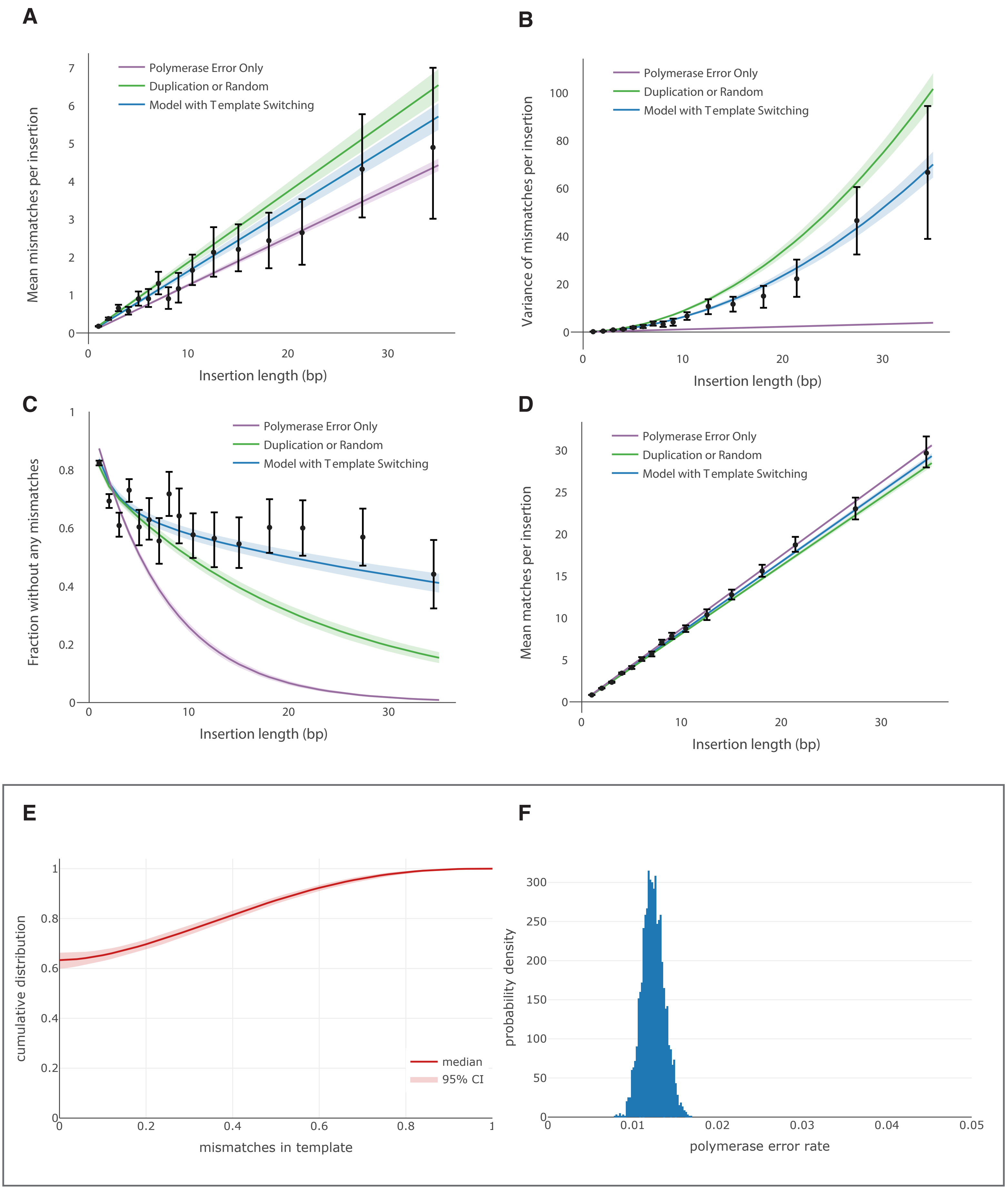

A
Mean mismatches per insertion
Insertion length (bp)
Polymerase Error Only
Duplication or Random
Model with Template Switching
B
Variance of mismatches per insertion
Insertion length (bp)
Polymerase Error Only
Duplication or Random
Model with Template Switching
C
Fraction without any mismatches
Insertion length (bp)
Polymerase Error Only
Duplication or Random
Model with Template Switching
D
Mean matches per insertion
Insertion length (bp)
Polymerase Error Only
Duplication or Random
Model with Template Switching
E
cumulative distribution
mismatches in template
median
95% CI
F
probability density
polymerase error rate

Fig. S10.

Modelling the provenance of indels in recombination hotspots. (A-D) Mean number of mismatches, variance in the number of mismatches, the proportion of sequences without any mismatch (perfect side-by-side duplications) and the mean number of matching bases per insertion under a model of random polymerase errors only (purple), random polymerase errors + choice of template (green) and random polymerase errors + choice of template + template-switching (blue). The black points are the observed values, with the error bars estimated using bootstrap resampling. For longer insertions (length>10bp) we bin insertions of similar lengths so there are at least 100 observations at each point. The shaded area around the model fit lines is the 95% confidence interval of the posterior distribution. **(E-F)** Inference from the model that permits template switching. **(E)** Cumulative distribution of the proportion of mismatches in the inserted sequence relative to the correct template (not including polymerase errors, i.e., 1-h where h is the homology between them). Shaded area shows the 95% confidence interval of the posterior distribution of the fitted model.
(F) Posterior distribution of the polymerase error rate from the model.

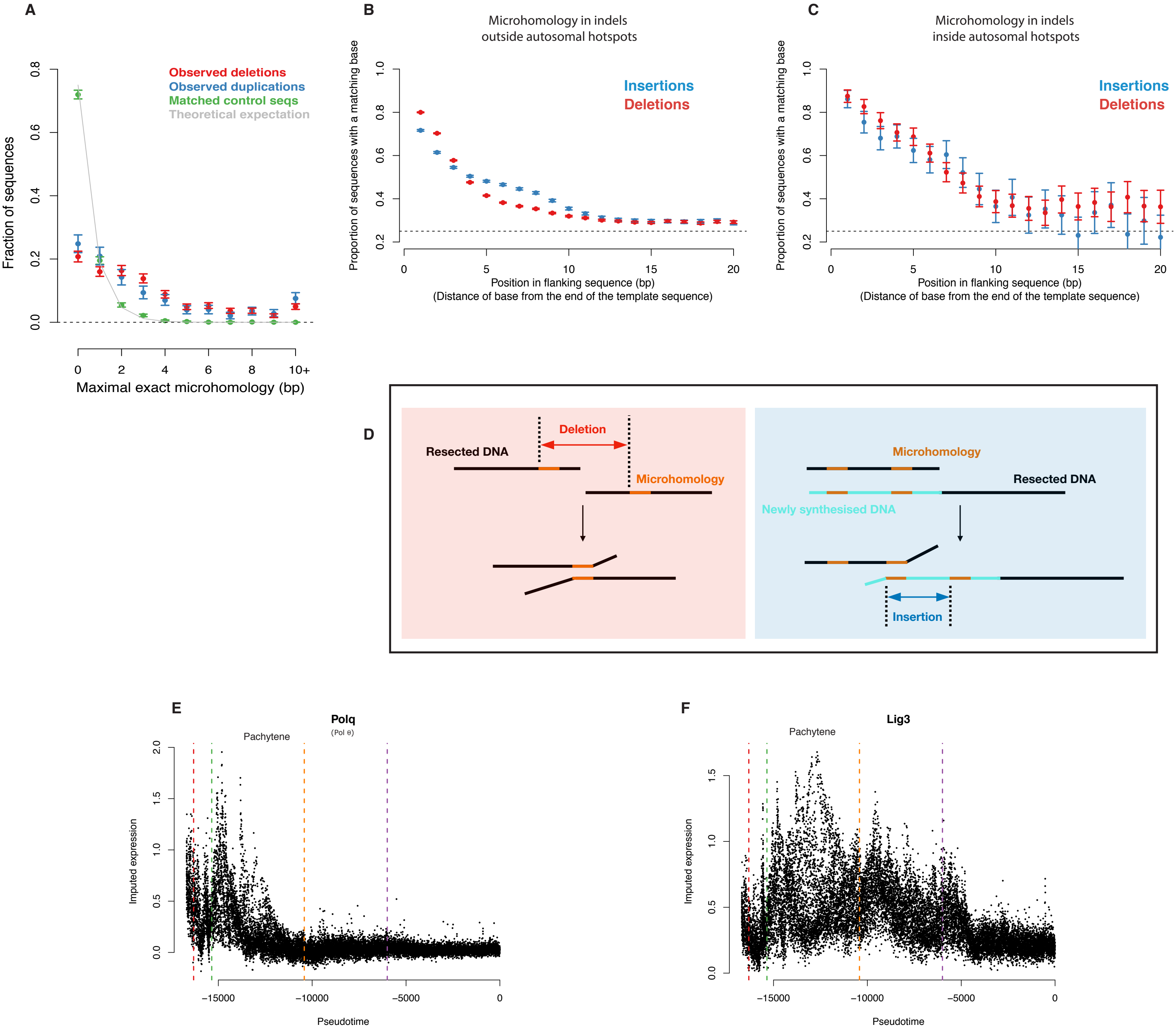
A
Observed deletions
Observed duplications
Matched control seqs
Theoretical expectation
Fraction of sequences
Maximal exact microhomology (bp)
B
Microhomology in indels
outside autosomal hotspots
Insertions
Deletions
Proportion of sequences with a matching base
Position in flanking sequence (bp)
(Distance of base from the end of the template sequence)
C
Microhomology in indels
inside autosomal hotspots
Insertions
Deletions
Proportion of sequences with a matching base
Position in flanking sequence (bp)
(Distance of base from the end of the template sequence)
D
Deletion
Resected DNA
Microhomology
Microhomology
Resected DNA
Newly synthesised DNA
Insertion
E
Polq
(Pol θ)
Pachytene
Imputed expression
Pseudotime
F
Lig3
Pachytene
Imputed expression
Pseudotime

Fig. S11. Provenance of indels originating in recombination hotspots. (A) Proportion of autosomal indels of length ≥10 bp in unique DNA that have an x-base pair microhomology between the deleted sequence and its flanking DNA (red, n=2217), between perfectly duplicated sequence and its flanking DNA (blue, n=822), matched control sequences for each deletion (± 50 bp) (green, n=4434), and theoretical expectation (grey). Indels with a breakpoint within 100 bp of hotspot centres were included. **(B)** Proportion of sequences in which the deleted (red) or inserted (blue) sequence matches its flanking DNA at base position x (see Fig. 7). Sequences >=5 bp in length and >=5 kb from hotspot centres were included. Error bars show 2 standard errors in the estimate of the mean **(C)** As (B) but the distribution inferredto be due to meiotic breaks (see Fig. 7 for details). **(D)** Model for microhomology-mediated end joining by DNA Polymerase θ (TMEJ) can generate deletions as well as insertions. Deletions (left) arise through pairing between segments of microhomology between resected strands. Insertions (right) arise through sites where DNA re-synthesis has already been initiated. A segment of DNA has been re-synthesized and extended, potentially using the homologue or sister chromatid as template. However, instead of re-annealing accurately, the re-synthesized DNA pairs ectopically with the complementary strand through a segment of microhomology. **(E)** Time-course of expression of Polq (Polθ) in mouse testis cells imputed via single-cell RNA-seq data. Dashed lines mark approximate transition points in meiotic stages from left to right: Spermatogonia, Leptotene/Zygotene, Pachytene, Meiotic divisions, Spermiogenesis. DNA break induction commences in Leptotene and most breaks are repaired by the end of Pachytene.
(F) As (E) but for Lig3. LIG3 operates downstream of POLθ in TMEJ.

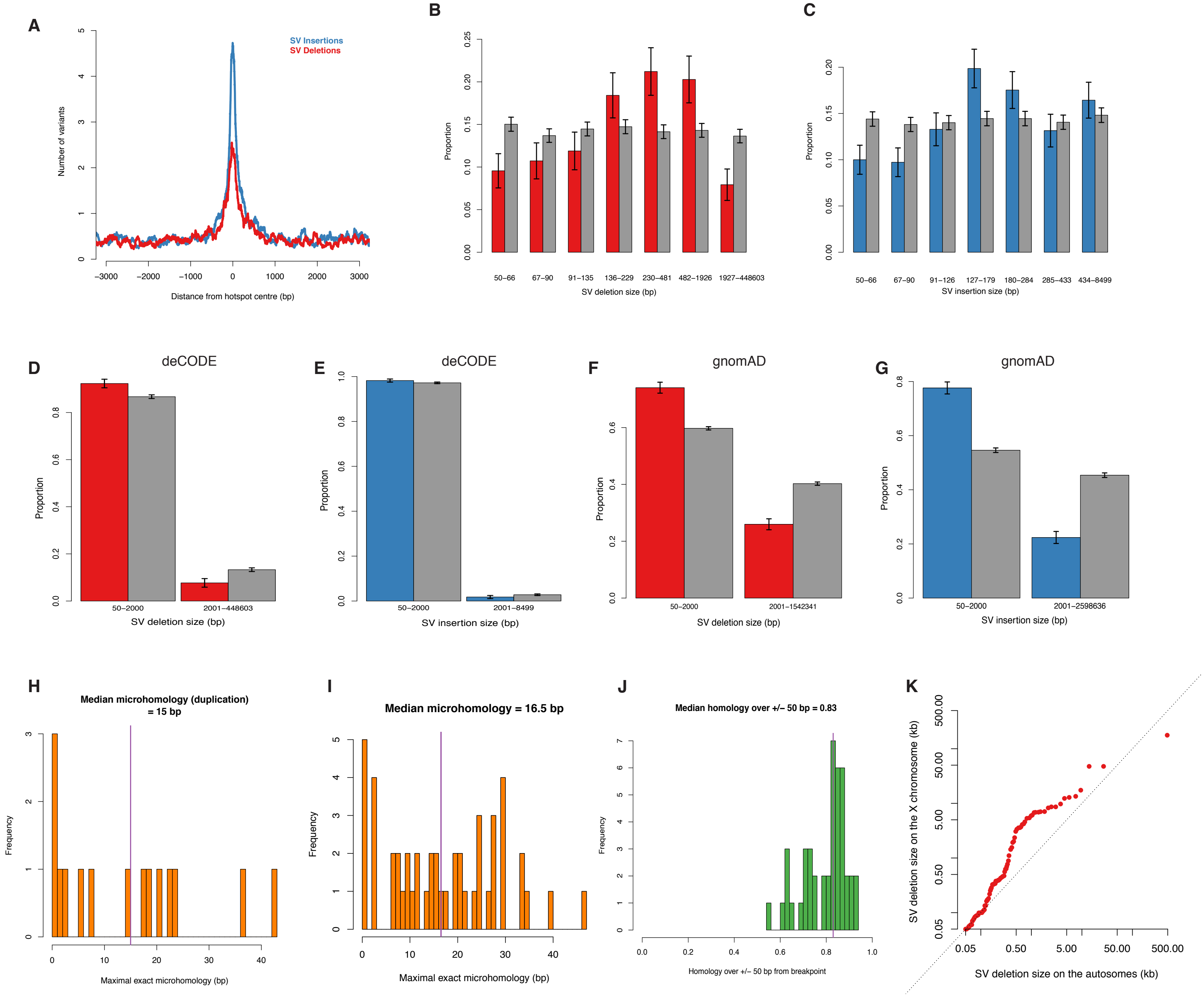
A
SV Insertions
SV Deletions
Number of variants
Distance from hotspot centre (bp)
B
Proportion
50−66
67−90
91−135
136−229
230−481
482−1926
1927−448603
SV deletion size (bp)
C
Proportion
50−66
67−90
91−126
127−179
180−284
285−433
434−8499
SV insertion size (bp)
D
deCODE
Proportion
50−2000
2001−448603
SV deletion size (bp)
E
deCODE
Proportion
50−2000
2001−8499
SV insertion size (bp)
F
gnomAD
Proportion
50−2000
2001−1542341
SV deletion size (bp)
G
gnomAD
Proportion
50−2000
2001−2598636
SV insertion size (bp)
H
Median microhomology (duplication)
= 15 bp
Frequency
Maximal exact microhomology (bp)
I
Median microhomology = 16.5 bp
Frequency
Maximal exact microhomology (bp)
J
Median homology over +/− 50 bp = 0.83
Frequency
Homology over +/− 50 bp from breakpoint
K
SV deletion size on the X chromosome (kb)
SV deletion size on the autosomes (kb)

Fig S12. Provenance of SVs originating in recombination hotspots. (A) Comparison of the absolute number of SV deletions and insertions in autosomal hotspots (100 bp smoothing, hotspot-proximal breakpoint shown). In other words, as Fig. 3C, but without background correction or inference of per-DSB mutation rate. **(B)** Proportion of deCODE SV deletion sizes inside (red) and outside (grey) hotspots. SVs outside hotspots had their hotspot-proximal breakpoint between 5kb and 10kb away and were divided into bins such that the total number of SVs in each bin was approximately the same (grey). The proportion of SVs with a breakpoint within 100 bp of hotspot centres is shown for each bin (red). **(C)** As (B) but for insertions (blue). **(D)** As (B) but with two size bins **(E)** As (D) but for insertions **(F)** As (D) but for gnomAD data. Note that gnomAD-SV calls are based on short-read sequencing, which has lower power to detect shorter variants that the long-read sequencing approach in deCODE-SV. Nevertheless, the proportions can be compared between the inside- and outside- hotspot sets and the odds ratio is similar for gnomAD and deCODE (see Supplementary Materials for details). **(G)** As (F) but for insertions **(H)** As Fig. 8B but for duplications without error. Amongst 98 SV insertions in deCODE-SV that were smaller than 2kb, only 15 were exact duplications. The maximal error-free microhomology with the flanking sequence is shown for those cases. Other insertions could not be assessed due to uncertainty in breakpoint location. **(I)** Histogram of the maximal error-free microhomology between the deleted sequence and its flanking sequence in SV deletions longer than 400 bp such that both breakpoints are in Alu elements. The hotspot-proximal breakpoint is within 100 bp of hotspot centres (n=54). The breakpoint was uncertain in complex SVs (n=6), which were excluded. **(J)** As (I) but histogram of overall homology in the region +/- 50 bp of breakpoints in SVs. Alu elements in 10 SVs failed to align with each other at this scale. **(K)** Quantile-quantile plot of SV deletion sizes on the X chromosome relative to the autosomes.

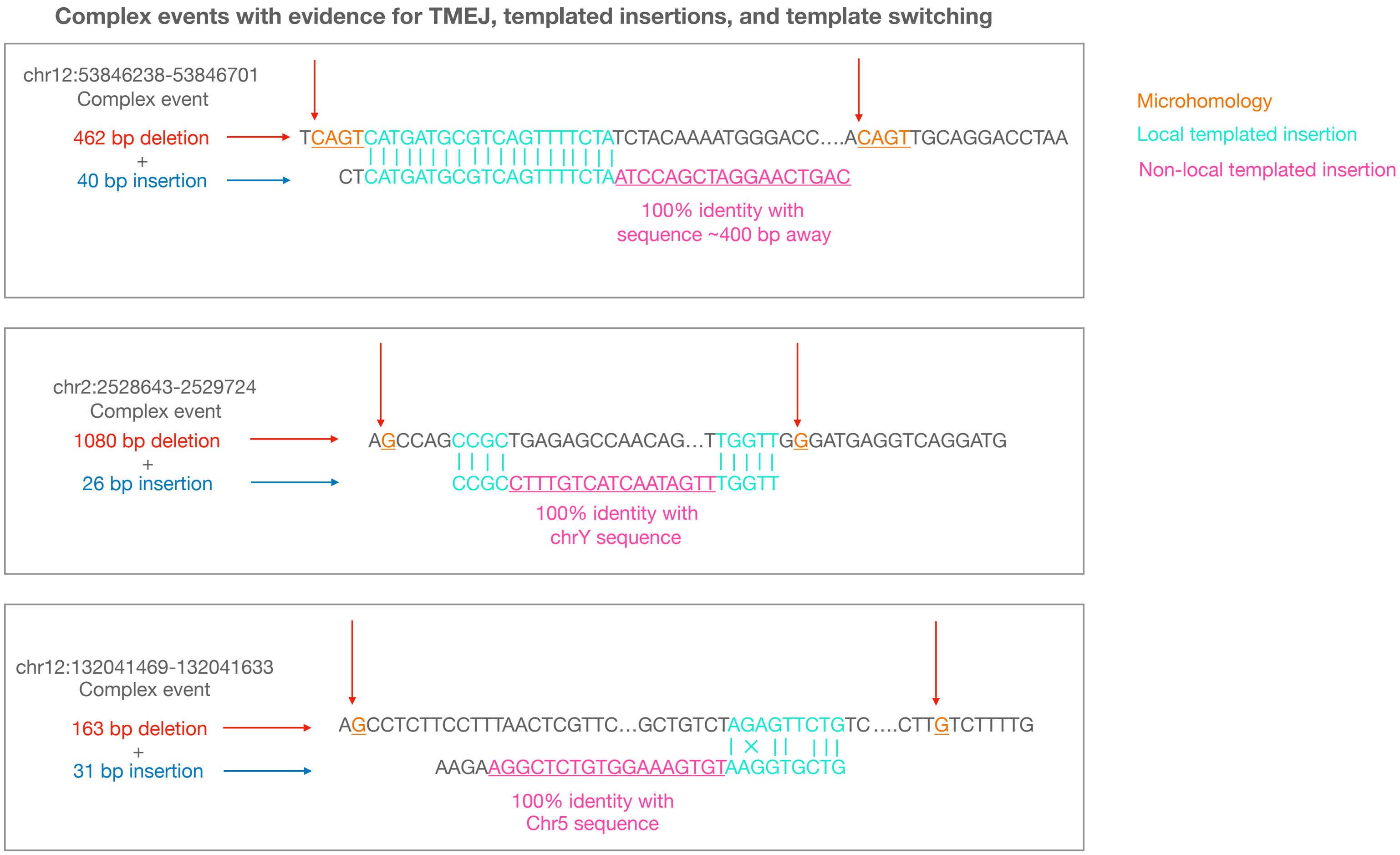

Fig. S13. Examples of complex SVs. In order to assess the underlying mechanisms generating SVs, we considered examples of complex SVs, i.e., those which contained both deletions and insertions. Specifically, we considered SVs wherein the inserted sequence was long enough for its sequence to be mapped to the genome with high mapping quality. Deletion breakpoints are marked with red arrows and microhomology at breakpoints is shown in orange. Inserted sequence homeologous with sequence close to a breakpoint is shown in blue and inserted sequence with homology to distal sequences (identified through BLAT) are shown in pink.

Whilst templated insertions are less common than not with TMEJ, they are nevertheless a more specific signature of TMEJ [Ramsden et al 2022, Schimmel et al 2019]. Templated insertions reflect one or more aborted rounds of synthesis and are associated with shorter homologies than is typical for TMEJ without templated insertions. [Ramsden et al 2022].

	De novo single-base substitutions (deCODE)	Unique DNA Insertions (All sizes)	Unique DNA Deletions (All sizes)	Unique DNA Insertions (10 bp+)	Unique DNA Deletions (10 bp+)	Tandem Repeat Insertions (All sizes)	Tandem Repeat Deletions (All sizes)
Model 1.							
Hotspot intensity	$1x10^{-13}$	$1x10^{-28}$	$9x10^{-62}$	$3x10^{-6}$	$1x10^{-11}$	$1x10^{-7}$	$1x10^{-4}$
TR copy number						$2x10^{-5}$	$2x10^{-8}$
Model 2.							
Hotspot intensity	$4x10^{-13}$	$5x10^{-26}$	$1x10^{-52}$	$5x10^{-6}$	$1x10^{-9}$	$2x10^{-7}$	$2x10^{-4}$
Background mutation rate	$2x10^{-5}$	$5x10^{-9}$	$2x10^{-65}$	0.12	$3x10^{-34}$	0.60	0.22
TR copy number						$8x10^{-4}$	$3x10^{-5}$
Model 3.							
Hotspot intensity	$3x10^{-12}$	$2x10^{-20}$	$9x10^{-41}$	$1x10^{-5}$	$1x10^{-8}$	$1x10^{-5}$	$5x10^{-3}$
Background mutation rate	$3x10^{-5}$	$9x10^{-8}$	$8x10^{-62}$	0.45	$2x10^{-30}$	0.68	0.46
Telomeric hotspot (within 1 Mb)	0.02	$7x10^{-8}$	$2x10^{-11}$	$8x10^{-10}$	$5x10^{-5}$	$5x10^{-3}$	$2x10^{-4}$
TR copy number						$1x10^{-4}$	$9x10^{-7}$

Table S1. Results of modelling the number of point and indel mutations in recombination hotspots in unique and tandem repeat DNA (See supplementary text for details).

Table S2. (separate file)
Predicted loss-of-function and high-impact SVs and indels (n=1,606) that are likely to have arisen as a result of the recombination machinery (p>0.5) (GRCh38 coordinates). They impact 958 genes and the genes with the most variants (n>=10 each) include *MUC4* (Lung cancer), *SLC19A1* (Megaloblastic anaemia), *PRDM9* (Meiotic recombination), *ARHGDIA* (Nephrotic syndrome), and *WNK1* (Hereditary neuropathy). The mutations reported in the main text are the ones most likely to have arisen as a result of meiotic break repair (average p=0.8, n=278). The variant types are insertions (INS), deletions (DEL), and complex SVs that may have both deleted and inserted sequence (CPX).

Table S3. (separate file)
Genes on the autosomes and the X chromosome with statistically significant excess of pathogenic mutations in ClinVar in hotspot-overlapping exons relative to other exons (p-value threshold of 0.05 after Bonferroni correction for 514 tests). Of the 81 genes reported here, 5 also appeared in Table S2. These are *CNGB1* (Retinitis Pigmentosa), *COL4A3* (Alport syndrome), *COL6A1* (Congenital muscular dystrophy), *MFRP* (Microphthalmia), *RP1L1* (Macular dystrophy).

Data S1. (separate file)
DMC1 hotspots for PRDM9 A-like alleles.
